# Effect of data leakage in brain MRI classification using 2D convolutional neural networks

**DOI:** 10.1038/s41598-021-01681-w

**Published:** 2021-11-19

**Authors:** Ekin Yagis, Selamawet Workalemahu Atnafu, Alba García Seco de Herrera, Chiara Marzi, Riccardo Scheda, Marco Giannelli, Carlo Tessa, Luca Citi, Stefano Diciotti

**Affiliations:** 1 School of Computer Science and Electronic Engineering, University of Essex, Colchester, UK; 2 Department of Electrical, Electronic, and Information Engineering “Guglielmo Marconi”, University of Bologna, Via dell’Università 50, 47521 Cesena, Italy; 3 Unit of Medical Physics, Pisa University Hospital “Azienda Ospedaliero-Universitaria Pisana”, Pisa, Italy; 4 Division of Radiology, Versilia Hospital, Azienda USL Toscana Nord Ovest, Lido di Camaiore, LU Italy

**Keywords:** Biomedical engineering, Neurodegenerative diseases

## Abstract

In recent years, 2D convolutional neural networks (CNNs) have been extensively used to diagnose neurological diseases from magnetic resonance imaging (MRI) data due to their potential to discern subtle and intricate patterns. Despite the high performances reported in numerous studies, developing CNN models with good generalization abilities is still a challenging task due to possible data leakage introduced during cross-validation (CV). In this study, we quantitatively assessed the effect of a data leakage caused by 3D MRI data splitting based on a 2D slice-level using three 2D CNN models to classify patients with Alzheimer’s disease (AD) and Parkinson’s disease (PD). Our experiments showed that slice-level CV erroneously boosted the average slice level accuracy on the test set by 30% on Open Access Series of Imaging Studies (OASIS), 29% on Alzheimer’s Disease Neuroimaging Initiative (ADNI), 48% on Parkinson’s Progression Markers Initiative (PPMI) and 55% on a local de-novo PD Versilia dataset. Further tests on a randomly labeled OASIS-derived dataset produced about 96% of (erroneous) accuracy (slice-level split) and 50% accuracy (subject-level split), as expected from a randomized experiment. Overall, the extent of the effect of an erroneous slice-based CV is severe, especially for small datasets.

## Introduction

Deep learning has become a popular class of machine learning algorithms in computer vision and has been successfully employed in various tasks, including multimedia analysis (image, video, and audio analysis), natural language processing, and robotics^1^. In particular, deep convolutional neural networks (CNNs) hierarchically learn high-level and complex features from input data, hence eliminating the need for handcrafting features, as in the case of conventional machine learning schemes^2^.

The application of these methods in neuroimaging is rapidly growing (see Greenspan et al.^3^ and Zaharchuk et al.^4^ for reviews). Several studies employed deep learning methods for image improvement and transformation^5–10^. Other studies performed lesion detection and segmentation^11–13^ and image-based diagnosis using different CNNs architectures^14,15^. Deep learning has also been applied to more complex tasks, including identifying patterns of disease subtypes, determining risk factors, and predicting disease progression (see, e.g., Zaharchuk et al.^4^ and Davatzikos^16^ for reviews). Early works applied stacked auto encoders^14,17,18^ and deep belief networks^19^ to classify neurological patients from healthy subjects using data collected from different neuroimaging modalities, including magnetic resonance imaging (MRI), positron emission tomography (PET), resting-state functional MRI (rsfMRI), and the combination of these modalities^20^.

Some authors reported very high accuracies in classifying patients with neurological diseases, such as Alzheimer’s disease (AD) and Parkinson’s disease (PD). For a binary classification of AD vs. healthy controls, Hon and Khan^21^ reported accuracy up to 96.25% using a transfer learning strategy. Sarraf et al.^22^ classified subjects as AD or healthy controls with a subject-level accuracy of 100% by adopting LeNet-5 and GoogleNet network architectures. In other studies, CNNs have been used for performing multi-class discrimination of subjects. Recently, Wu et al.^23^ adopted a pre-trained CaffeNet and achieved accuracy of 98.71%, 72.04%, and 92.35% for a three-way classification between healthy controls, stable mild cognitive impairment (MCI), and progressive MCI patients, respectively. In another work by Islam and Zhang^24^, an ensemble system of three homogeneous CNNs has been proposed, and average multi-class classification accuracy of 93.18% was found on the Open Access Series of Imaging Studies (OASIS) dataset. For the classification of PD, Esmaeilzadeh et al.^25^ classified PD patients from healthy controls based on MRI and demographic information (i.e., age and gender). With the proposed 3D model, they achieved 100% accuracy on the test set. In another study by Sivaranjini and Sujatha^26^, a pre-trained 2D CNN AlexNet architecture was used to classify PD patients vs. healthy controls, resulting in an accuracy of 88.9%.

Although excellent performances have been shown by using deep learning for the classification of neurological disorders, there are still many challenges that need to be addressed, including complexity and difficulty in interpreting the results due to highly nonlinear computations, non-reproducibility of the results, and data/information and, especially, data overfitting (see Vieira et al.^20^ and Davatzikos^16^ for reviews).

Overly optimistic results may be due to data leakage—a process caused by the use of information in the model training that is not expected to be available at prediction time. See Kaufman et al.^27^ for further details on a formal definition of data leakage. Data leakage can be due to a target (class label) leakage or incorrect data split. For example, data leakage may occur when feature selection is performed based on the whole dataset before cross-validation^28,29^. In this case, the target variable of samples in the test sets may be erroneously used for improving the learning process. Several cases may be related to an incorrect data split. For example, when the data augmentation step is performed before dividing the test set from the training data (late split), the augmented data generated from the same original image can be seen in both training and test data, leading to incorrect inflated performance^30^. Another form of train-test contamination that leads to data leakage is when the same test set is used to optimize the training hyperparameters and evaluate the model performance^29^. A different use of information not available at prediction time occurs using longitudinal data, when there is a danger of information leaking from the future to the past. A particularly insidious form of data leakage may occur when information about the target inadvertently leaks into the input data, for example the presence of a ruler, markings or treatment devices in a medical image may correlate with the class label^31–33^.

While concluding that data leakage leads to overly optimistic results will surprise few practitioners, we believe that the extent to which this is happening in neuroimaging applications is mostly unknown, especially in small datasets. As we completed this study, we became aware of independent research by Wen et al.^30^ that corroborates part of our conclusions regarding the problem of data leakage. They successfully suggested a framework for the reproducible assessment of AD classification methods. However, the architectures have not been trained and tested on smaller datasets typical of clinical practice, and they mainly employed hold-out model validation strategies rather than cross-validation (CV)—that gives a better indication of how well a model performs on unseen data^34,35^. Moreover, the authors focused on illustrating the effect of data leakage on the classification of AD patients only.

Unfortunately, the problem of data leakage incurred by incorrect data split is not only limited within the area of AD classification but can also be seen in various other neurological disorders. It is more common to observe the data leakage in 2D architectures, yet some forms of data leakage, such as late split, could be present in 3D CNN studies as well. Moreover, although deep complex classifiers are more prone to overfitting, also conventional machine learning algorithms may be affected by data leakage. A summary of these works with clear and potential data leakage is given in Tables 1 and 2, respectively. Other works with insufficient information to assess data leakage are reported in Table 3.

**Table 1:** Summary of the previous studies performing classification of neurological disorders using MRI and with clear data leakage (see also Supplementary Table S1 for a detailed description).

Disorder	References	Groups (number of subjects)	Machine learning model	Data split method	Type of data leakage	Accuracy (%)
AD/MCI	Gunawardena et al.^36^	AD-MCI-HC (36)	2D CNN	4:1 train/test slice-level split	Wrong split	96.00
	Hon and Khan^21^	AD-HC (200)	2D CNN (VGG16)	4:1 train/test slice-level split	Wrong split	96.25
	Jain et al.^37^	AD-MCI-HC (150)	2D CNN (VGG16)	4:1 train/test slice-level split	Late and wrong split	95.00
	Khagi et al.^38^	AD-HC (56)	2D CNN (AlexNet, GoogLeNet,ResNet50, new CNN)	6:2:2 train/validation/test slice-level split	Wrong split	98.00
	Sarraf et al.^22^	AD-HC (43)	2D CNN (LeNet-5)	3:1:1 train/validation/test slice-level split	Wrong split	96.85
	Wang et al.^39^	MCI-HC (629)	2D CNN	Data augmentation + 10:3:3 train/validation/test split by MRI slices	Wrong split and augmentation before split	90.60
	Puranik et al.^40^	AD/EMCI-HC (75)	2D CNN	17:3 train/test split by MRI slices	Wrong split	98.40
	Basheera et al.^41^	AD-MCI-HC (1820)	2D CNN	4:1 train/test split by MRI slices	Wrong split	90.47
	Nawaz et al.^42^	AD-MCI-HC (1726)	2D CNN	6:2:2 slice level split	Wrong split	99.89

*AD* Alzheimer’s disease, *HC* healthy controls, *MCI* mild cognitive impairment.

**Table 2:** Summary of the previous studies performing classification of neurological disorders using MRI and suspected to have potential data leakage (see also Supplementary Table S2 for a detailed description).

Disorder	References	Groups (number of subjects)	Machine learning model	Data split method	Type of data leakage	Accuracy (%)
AD/MCI	Farooq et al.^43^	AD-MCI-LMCI-HC (355)	2D CNN (GoogLeNet and modified ResNet)	3:1 train/test (potential) slice-level split	Wrong split	98.80
	Ramzan et al.^44^	HC-SMC- EMCI-MCI-LMCI-AD (138)	2D CNN (ResNet-18)	7:2:1 train/validation/test (potential) slice-level split	Wrong split	100
	Raza et al.^45^	AD-HC (432)	2D CNN (AlexNet)	4:1 train/test (potential) slice-level split	Wrong split	98.74
	Pathak et al.^46^	AD-HC (266)	2D CNN	3:1 (potential) slice level split	Wrong split	91.75
ASD	Libero et al.^47^	ASD-TD (37)	Decision tree	unclear	Entire data set used for feature selection	91.90
	Zhou et al.^48^	ASD-TD/HC (280)	Random tree classifier	4:1 train/test split	Entire data set used for feature selection	100
PD	Sivaranjini, et al.^26^	PD-HC (182)	2D CNN	4:1 train/test split by MRI slices	Wrong split	88.90
TBI	Lui et al.^49^	TBI-HC (47)	Multilayer perceptron	tenfold CV	Entire data set used for feature selection	86.00
Brain tumor	Hasan et al.^50^	Tumor-HC (600)	MGLCM + 2D CNN + SVM	tenfold CV	Wrong split and entire data set used for feature selection	99.30

*AD* Alzheimer’s disease, *ASD* Autism spectrum disorder, *EMCI* early mild cognitive impairment, *HC* healthy controls, *LMCI* late mild cognitive impairment, *MCI* Mild cognitive impairment, *MGLCM* modified gray level co-occurrence matrix, *PD* Parkinson’s disease, *SMC* subjective memory concerns, *TBI* traumatic brain injury, *TD* typically developing.

**Table 3:** Summary of the previous studies performing classification of neurological disorders using MRI and that provide insufficient information to assess data leakage (see also Supplementary Table S3 for a detailed description).

Disorder	References	Groups (number of subjects)	Machine learning model	Data split method	Accuracy (%)
AD/MCI	Al-Khuzaie et al.^51^	AD-HC (240)	2D CNN	(Potential) slice-level split	99.30
	Wu et al.^23^	AD-HC (457)	2D CNN	Data augmentation + 2:1 train/test split by MRI slices	97.58

*AD* Alzheimer’s disease, *HC* healthy controls, *MCI* mild cognitive impairment.

In this study, we addressed the issue of data leakage in one of the most common classes of deep learning models, i.e., 2D CNNs, caused by incorrect dataset split of 3D MRI data. Specifically, we quantified the effect of data leakage on CNN models trained on different datasets of T_1_-weighted brain MRI of healthy controls and patients with neurological disorders using a nested CV scheme with two different data split strategies: (a) subject-level split, avoiding any form of data leakage and (b) slice-level split, in which different slices of the same subject are contained both in the training and the test folds (thus data leakage will occur). We focused our attention on both large (about 200 subjects) and small (about 30 subjects) datasets to evaluate a possible increase of performance overestimation when a smaller dataset was used, as is often the case in clinical practice. This paper expands on the preliminary results by Yagis et al.^52^, offering a broader investigation on the issue. In particular, we performed the classification of AD patients using the following datasets: (1) OASIS-200, consisting of randomly sampled 100 AD patients and 100 healthy controls from the OASIS-1 study^53^, (2) ADNI, including 100 AD patients and 100 healthy controls randomly sampled from Alzheimer’s Disease Neuroimaging Initiative (ADNI)^54^, and (3) OASIS-34, composed of 34 subjects (17 AD patients and 17 healthy controls) randomly selected from the OASIS-200 dataset. Given that the performance of a model trained on a small sample dataset could depend on the selected samples, we created ten instances of the OASIS-34 dataset by randomly sampling from the OASIS-200 dataset ten times independently. The subject IDs included in each instance are found in Supplementary Table S4. Moreover, we generated a different dataset, called OASIS-random, where, for each subject of the OASIS-200 dataset, a fake random label of either AD patient or healthy control was assigned. In this case, the image data had no relationship with the assigned labels. Besides, we included two T_1_-weighted images datasets of patients with de-novo PD: PPMI, including 100 de-novo PD patients and 100 healthy controls randomly chosen from the public Parkinson’s Progression Markers Initiative (PPMI) dataset^55^, and Versilia, a small-sized private clinical dataset of 17 patients with de-novo PD and 17 healthy controls. A detailed description of each dataset has been reported in the “Materials and methods” section.

## Results

For AD classification, accuracy on the test set, using subject-level CV, was below 71% for large datasets (OASIS-200 and ADNI), whereas they were below 59% for smaller datasets (OASIS-34). Regarding de novo PD classification, they were around 50% for both large (PPMI) and small (Versilia) datasets. Conversely, slice-level CV erroneously produced very high classification accuracies on the test set in all datasets (higher than 94% and 92% on large and small datasets, respectively), leading to deceptive, over-optimistic results (Table 4).

**Table 4:** Mean slice-level accuracy on the training and test set of the outer CV over fivefold nested CV has been reported for three 2D CNN models (see “Materials and methods” section), all datasets, and two data split methods (slice-level and subject-level).

Dataset	Network architecture	Training set accuracy (%)		Test set accuracy (%)		
		Subject-level split	Slice-level split	Subject-level split	Slice-level split	Difference
OASIS-200	VGG16-v1	95.93	99.85	66.0	94.18	28.18
	VGG16-v2	95.13	100	66.13	96.99	30.86
	ResNet-18	100	100	68.87	98.96	30.1
OASIS-34	VGG16-v1	88.94	100	54.35	99.19	44.84
	VGG16-v2	96.94	100	54.34	99.33	44.99
	ResNet-18	100	100	57.49	98.96	41.47
OASIS-random	VGG16-v1	63.38	100	53.37	95.93	42.56
	VGG16-v2	69.17	100	49.25	94.81	45.56
	ResNet-18	84.49	99.09	50.8	93.74	42.94
ADNI	VGG16-v1	91.09	100	70.12	95.31	25.19
	VGG16-v2	80.49	100	66.49	95.24	28.75
	ResNet-18	100	100	68.68	96.87	30.19
PPMI	VGG16-v1	76.8	100	48.24	93.99	45.75
	VGG16-v2	73.19	100	46.93	94.37	47.44
	ResNet-18	100	100	48.06	96.12	44.06
Versilia	VGG16-v1	99.72	100	53.86	95.97	42.11
	VGG16-v2	76.89	100	42.97	97.8	54.83
	ResNet-18	99.90	95.13	51.36	92.63	41.27

The difference between accuracy using slice-level and subject-level split in the test set has also been reported.

The worst-case stemmed from the randomly labeled OASIS dataset, which resulted in a model with unacceptably high performances (accuracy on the test set more than 93%) using slice-level CV, whereas classification results obtained using a subject-level CV were about 50%, in accordance with the expected outcomes for a balanced dataset with completely random labels.

## Discussion

In this study, we quantitatively assessed the extent of the overestimation of the model’s classification performance caused by an incorrect slice-level CV, which is unfortunately adopted in neuroimaging literature (see Tables 1, 2, 3). More specifically, we showed the performance of three 2D CNN models (two VGG variants and one ResNet-18, see “Materials and methods” section) trained with subject-level and slice-level CV data splits to classify AD and PD patients from healthy controls using T_1_-weighted brain MRI data. Our results revealed that pooling slices of MRI volumes for all subjects and then dividing randomly into training and test set leads to significantly inflated accuracies (in some cases from barely above chance level to about 99%). In particular, slice-level CV erroneously increased the average slice level accuracy on the test set by 40–55% on smaller datasets (OASIS-34 and Versilia) and 25–45% on larger datasets (OASIS-200, ADNI, PPMI). Moreover, we also conducted an additional experiment in which all the labels of the subjects were fully randomized (OASIS-random dataset). Even under such circumstances, using the slice-level split, we achieved an erroneous 95% classification accuracy on the test set with all models, whereas we found 50% accuracy using a subject-level data split, as expected from a randomized experiment. This large (and erroneous) increase in performance could be due to the high intra-subject correlation among T_1_-weighted slices, resulting in a similar information content present in slices of the same subject^56^.

In AD classification, three previous studies^21,22,43^, using similar deep networks (VGG16, ResNet-18 and LeNet-5, respectively), reported higher classification accuracies (92.3%, 98.0% and 96.8%, respectively) than ours. However, there is a strong indication that these performances are massively overestimated due to a slice-level split. In particular, in one of these works^21^, the presence of data leakage was further corroborated by the source code accompanying the paper and confirmed by our data. In fact, when we used the same dataset of Hon and Khan^21^ (OASIS-200 dataset), our VGG16 models achieved only 66% classification accuracy with subject-level split, whereas they boosted to about 97% with a slice-level split. Similar findings were presented by Wen et al.^30^, who used an ADNI dataset with 330 healthy controls and 336 AD patients. Indeed, using baseline data, they reported a 79% of balanced accuracy in the validation set with a subject-level split which increased up to 100% with a slice-level split.

One of the main issues in the classification of neurological disorders using deep learning is data scarcity^57^. Not only because labeling is expensive but also because privacy reasons and institutional policies make acquiring and sharing large sets of labeled imaging data even more challenging^58^. To show the impact of data size on model performance, we created 10 small subsets from the OASIS dataset (OASIS-34 datasets). As expected, when we reduced the data, we obtained lower classification accuracies with all the networks using the subject-level data split method. However, when the slice-level method was used, the models erroneous achieved better results on OASIS-34 than on the OASIS-200 dataset. Similarly, models trained on the Versilia dataset (34 subjects) produced inflated results with the slice-level split. Overall, these results indicate that data leakage is highly relevant, especially when small datasets are used, which may, unfortunately, be common in clinical practice.

It is well-known that data leakage leads to inflating performance—and this phenomenon is not specific to brain MRI or deep learning, but it can occur in any machine learning system. Nevertheless, the degree of overestimation quantified through our experiments was surprising. Unfortunately, in the literature, the precise application of CV is frequently not well-documented, and the source code is not available, although we have observed these issues mostly in manuscripts that were either not peer-reviewed or not rigorously peer-reviewed (see Tables 1, 2, 3). Overall, this situation leaves the neuroimaging community unable to trust the (sometimes) promising results published. Regardless of the network architecture, the number of subjects, and the level of complexity of the classification problem, all experiments that applied slice-level CV yielded very high classification accuracies on the test set as a result of incorporating different slices of the same subject in both the training and test sets. Considering classifications on 2D MRI images, we showed that it is crucial that the CV split be done based on the subject-level to prevent data leakage and get trustable results. This assures that the training and validation sets to be completely independent and confirms that no information is leaking from the test set into the training set during the development of the model. Additionally, employing 3D models for 3D data with subject-level train-test split should be encouraged as 2D models do not effectively capture 3D features. The high computational complexity of 3D models may be tackled using image patches or sub-images, and parallel processing on multiple GPUs, or, in some cases, by image downsampling.

With recent advances in machine learning, more and more people are becoming interested in applying these techniques to biomedical imaging, and there is a real and growing risk that not all researchers pay sufficient attention to this serious issue. We also emphasize the need to document how the CV is implemented, the architecture used, how the different hyperparameter choices/tunings are made and include their values where possible. Besides, we advocate reproducibility and encourage the community to take a step towards transparency in deep/machine learning in medical image analysis by publicly releasing code, including containers and a link to open datasets^59^. Moreover, a blind evaluation on external test sets—i.e., within open challenges—is highly recommended.

One limitation of this study is due to the substantial overfitting we observed while applying a subject-level split for training our models. This overfitting is manifested by the very high accuracy in training sets compared to that observed in test sets (Table 4). Focussing our efforts on alleviating overfitting may have improved performance in the test set, thus reducing the extent of the faulty boost due to the slice-level split. Moreover, in this study, we have not assessed all data leakage types, including late split and hyperparameters optimization in the test set—that may also be present in 3D CNN studies. We have found evidence of all these data leakage issues in the recent literature (see Tables 1, 2, 3), and we plan to quantify their effect in our future work systematically.

In conclusion, training a 2D CNN model for analyzing 3D brain image data must be performed using a subject-level CV to prevent data leakage. The adoption of slice-based CV results in very optimistic model performances, especially for small datasets, as the extent of the overestimation due to data leakage is severe.

## Materials and methods

### Datasets

In this study, we adopted the scans collected by three public and international datasets of T_1_-weighted images of patients with AD (the OASIS dataset^53^ and the ADNI dataset^54^) and de-novo PD (the PPMI dataset^55^). An additional private de-novo PD dataset, namely the Versilia dataset, has also been used. A summary of the demographics of the datasets used in this study is shown in Table 5. In the following sections, a detailed description of all datasets will be reported.

**Table 5:** Demographic features of subjects belonging to OASIS-200, ADNI, PPMI, and Versilia datasets.

Dataset	Patients	Healthy controls
**OASIS-200**		
Number of subjects	100	100
Age (range, years)	62–96	59–94
Age (mean ± SD, years)	76.70 ± 7.10	75.50 ± 9.10
Gender (women/men)	59/41	73/27
**ADNI**		
Number of subjects	100	100
Age (range, years)	56–89	58–95
Age (mean ± SD, years)	74.28 ± 7.96	75.04 ± 7.11
Gender (women/men)	44/56	52/48
**PPMI**		
Number of subjects	100	100
Age (range, years)	34–82	31–83
Age (mean ± SD, years)	61.71 ± 9.99	61.91 ± 11.52
Gender (women/men)	40/60	36/64
**Versilia**		
Number of subjects	17	17
Age (range, years)	48–78	54–77
Age (mean ± SD, years)	64 ± 7.21	64.00 ± 7.00
Gender (women/men)	4/13	5/12

The same information for the OASIS-34 datasets has been reported in Supplementary Table S5.

*AD* Alzheimer’s disease, *ADNI* Alzheimer’s Disease Neuroimaging Initiative, *OASIS* open access series of imaging studies, *PD* Parkinson’s disease, *PPMI* Parkinson’s Progression Markers Initiative, *SD* standard deviation.

#### OASIS-200, OASIS-34, and OASIS-random datasets

We have used the T_1_-weighted images of 100 AD patients [(59 women and 41 men, age 76.70 ± 7.10 years, mean ± standard deviation (SD)] and 100 healthy controls (73 women and 27 men, age 75.50 ± 9.10 years, mean ± SD) from the OASIS-1 study—a cross-sectional cohort of the OASIS brain MRI dataset^53^, freely available at https://www.oasis-brains.org/. In particular, we have employed the same scans that were previously selected by other authors^21^. We called this dataset OASIS-200. The subject identification numbers (IDs) and demographics of these subjects were specified in Supplementary Table S6. No significant difference in age (p = 0.15 at t-test) was found between the two groups, while a significant (borderline) difference in gender was observed (p = 0.04 at χ^2^-test).

In OASIS-1, AD diagnosis, as well as the severity of the disease, were evaluated based on the global Clinical Dementia Rating (CDR) score derived from individual CDR scores for the domains memory, orientation, judgment and problem solving, function in community affairs, home and hobbies, and personal case^60,61^. Subjects with a global CDR score of 0 have been labeled as healthy controls, while scores 0.5 (very mild), 1 (mild), 2 (moderate), and 3 (severe) have been all labeled as AD.

All T_1_-weighted images have been acquired on a 1.5 T MR scanner (Vision, Siemens, Erlangen, Germany), using a Magnetization Prepared Rapid Gradient Echo (MPRAGE) sequence in a sagittal plane [repetition time (TR) = 9.7 ms, echo time (TE) = 4.0 ms, flip angle = 10°, inversion time (TI) = 20 ms, delay time (TD) = 200 ms, voxel size = 1 mm × 1 mm × 1.25 mm, matrix size = 256 × 256, number of slices = 128]^53^.

#### ADNI dataset

We considered the T_1_-weighted MRI data of 100 AD patients (44 women and 56 men, age 74.28 ± 7.96 years, mean ± SD) and 100 healthy controls (52 women and 48 men, age 75.04 ± 7.11 years, mean ± SD). No significant difference in age (p = 0.24 at t-test) and gender (p = 0.26 at χ^2^-test) was found between the two groups. Alzheimer’s disease patients have been randomly chosen from the ADNI 2 dataset (available at http://adni.loni.usc.edu/)—acohort of ADNI that extends the work of ADNI 1 and ADNI-GO studies^54^. Led by Principal Investigator Michael W. Weiner, MD, ADNI was launched in 2003 to investigate if biological markers (such as MRI and PET) can be combined to define the progression of MCI and early AD. We have used MPRAGE T_1_-weighted MRI scans acquired by 3 T scanners [6 Siemens (Erlangen, Germany) MRI scanners and 6 Philips (Amsterdam, Netherlands) scanners] in a sagittal plane (voxel size = 1 mm × 1 mm × 1.2 mm). The image size of the T_1_-weighted data acquired from the Siemens and Philips scanners were 176 × 240 × 256 and 170 × 256 × 256, respectively. Since ADNI 2 is a longitudinal dataset, more than one scan was available for each subject. The first scan of each participant has been chosen to produce a cross-sectional dataset. Supplementary Table S7 provides subject IDs and the acquisition date of the specific scan used in our study. The MRI acquisition protocol for each MRI scanner can be found at http://adni.loni.usc.edu/methods/documents/mri-protocols/. In ADNI 2 dataset, subjects have been categorized as AD patients or healthy controls based on whether subjects have complaints about their memory and by considering a combination of neuropsychological clinical scores^54^.

#### PPMI dataset

We randomly selected 100 de-novo PD subjects (40 women and 60 men, age 61.71 ± 9.99, mean ± SD) and 100 healthy controls (36 women and 64 men, age 61.91 ± 11.52, mean ± SD) from the publicly available PPMI dataset (https://ida.loni.usc.edu/login.jsp?project=PPMI). No significant difference in age (p = 0.44 at t-test) and gender (p = 0.56 at χ^2^-test) was found between the two groups. The criterion used to recruit de-novo PD patients, and healthy controls were defined by Marek et al.^55^. Briefly, PD patients were selected within two years of diagnosis with a Hoehn and Yahr score < 3^62^, at least two of resting tremor, either bradykinesia or rigidity (must have either resting tremor or asymmetric bradykinesia) or a single asymmetric resting tremor or asymmetric bradykinesia and dopamine transporter (DAT) or vesicular monoamine transporter type 2 (VMAT-2) imaging showing a dopaminergic deficit. Healthy controls were free from any clinically significant neurological disorder^55^.

The T_1_-weighted scans were collected at baseline using MR scanners manufactured by Siemens (11 scanners at 3 T and five scanners at 1.5 T), Philips Medical Systems (10 scanners at 3 T and 11 scanners at 1.5 T), GE Medical Systems (11 scanners at 3 T and 24 scanners at 1.5 T) and another anonymous one (5 scanners at 1.5 T). We also found three subjects whose MRI protocol was missing. The details of the MRI protocols of all scanners can be found in Supplementary Table S8.

#### Versilia dataset

Seventeen (4 women and 13 men, age 64 ± 7.21 years, mean ± SD) patients with de-novo parkinsonian syndrome consecutively referred to a Neurology Unit to evaluate PD over a 24-month interval (from June 2012 to June 2014) were recruited in this dataset. More details about clinical evaluation can be found in Ref.^63^. Seventeen healthy controls (5 women and 12 men, age 64 ± 7 years, mean ± SD) with no history of neurological diseases and normal neurological examination were recruited as controls. No significant difference in age (p = 0.95 at t-test) and gender (p = 0.70 at χ^2^-test) was found between the two groups.

All subjects underwent high-resolution 3D T_1_-weighted imaging on a 1.5 T MR scanner system (Magnetom Avanto, software version Syngo MR B17, Siemens, Erlangen-Germany) equipped with a 12-element matrix radiofrequency head coil and SQ-engine gradients. The SQ-engine gradients had a maximum strength of 45 mT/m and a slew rate of 200 T/m/s. T_1_-weighted MR images were acquired with an axial high resolution 3D MPRAGE sequence with TR = 1900 ms, TE = 3.44 ms, TI = 1100 ms, flip angle = 15°, slice thickness = 0.86 mm, field of view (FOV) = 220 mm × 220 mm, matrix size = 256 × 256, number of excitations (NEX) = 2, number of slices = 176.

### T_1_-weighted MRI data preprocessing

**Figure 1:**
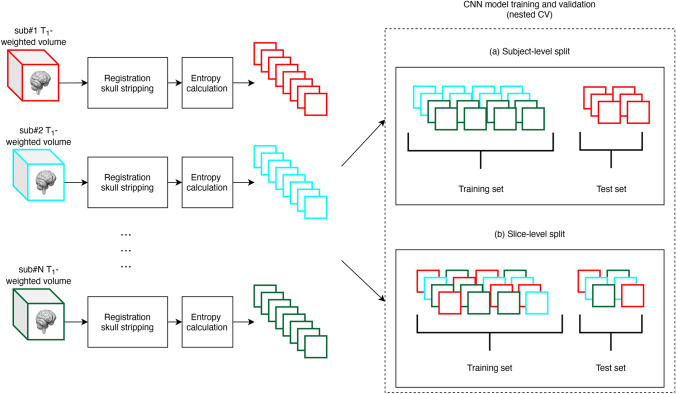
Schematic diagram of the overall T_1_-weighted MRI data processing and validation scheme. First, a preprocessing stage included co-registration to a standard space, skull-stripping and slices selection based on entropy calculation. Then, CNNs model’s training and validation have been performed on each dataset in a nested CV loop using two different data split strategies: (a) subject-level split, in which all the slices of a subject have been placed either in the training or in the test set, avoiding any form of data leakage; (b) slice-level split, in which all the slices have been pooled together before CV, then split randomly into training and test set.

All T_1_-weighted MRI data went through two preprocessing steps (see Fig. 1). In the first stage, co-registration to a standard template space and skull stripping were applied to re-align all the images and remove non-brain regions. In the second stage, a subset of axial images has been collected using an entropy-based slice selection approach.

#### Co-registration to a standard template space and skull stripping

For the OASIS datasets, we used publicly available preprocessed data (gain-field corrected, brain masked, and co-registration)^64^. Briefly, the brain masks from OASIS were obtained using an atlas-registration-based method, and their quality was controlled by human experts^53^, and each volume has been co-registered to the Talairach and Tournoux atlas. Each preprocessed T_1_-weighted volume had a data matrix size of 176 × 208 × 176 and a voxel size of 1 mm × 1 mm × 1 mm^64^.

For all other datasets, we have co-registered each individual T_1_-weighted volume to the MNI152 standard template space (at 1 mm voxel size—available in the FSL version 6.0.3 package) by using the SyN algorithm included in ANTs package (version 2.1.0) with default parameters^65^. Then, the brain mask of the standard template space has been applied to each co-registered volume. Each preprocessed T_1_-weighted volume had a data matrix size of 182 × 218 × 182 and a voxel size of 1 mm × 1 mm × 1 mm.

Supplementary Figure S1 online illustrates sample preprocessed T_1_-weighted slices from OASIS-200, ADNI, PPMI, and Versilia datasets.

#### Entropy-based slice selection

Each T_1_-weighted slice generally conveys a different amount of information. Given that we are interested in developing a 2D CNN model, we have performed a preliminary slice selection based on the amount of information. More specifically, for each T_1_-weighted volume, the Shannon entropy *E*_*S*_, representing the information content, was computed for each axial slice, as follows:

1 $${E}_{S}=\sum_{k}{p}_{k}{\text{log}}_{2}({p}_{k}),$$

where *k* is the number of grayscale levels in the slice and *p*_*k*_ is the probability of occurrence, estimated as the relative frequency in the image, for the gray level *k*. Then, for each T_1_-weighted volume, the slices were ordered in descending order based on their entropy scores, and, finally, we selected only the eight axial slices that showed the highest entropy^21^.

To be consistent with the input sizes of the proposed 2D CNN models, all slices were resized to 224 × 224 pixels by fitting a cubic spline between the 4-by-4 neighborhood pixels^66^. Voxel-wise feature standardization has also been applied to make training the CNNs easier and achieve faster convergence, i.e., for each voxel, an average value of all grayscale values within the brain mask has been subtracted and scaled by the standard deviation (within the brain mask).

### Model architectures

Since the number of subjects of each dataset may not be sufficient to train with high accuracy a 2D CNN model from scratch, we have used a machine learning technique called transfer learning that allows employing pre-trained models, i.e., model parameters previously developed for one task (source domain) to be transferred to target domain for weight initialization and feature extraction. In particular, CNN layers hierarchically extract features starting from the general low-level features to those specific to the target class, and, using transfer learning, the general low-level features can be shared across tasks. Notably, we used pre-trained VGG16^67^ and ResNet-18^68^ models in this study, as detailed in the following sections. The transfer learning approach and VGG16 architectures used in this study are similar to those employed in Ref.^21^ as their results triggered our investigation of data leakage.

#### VGG16-based models

VGG16 is one of the most influential architectures which explores network depth with very small (3 × 3) convolution filters stacked on top of each other. VGG16 consists of five convolutional blocks, with alternating convolutional and pooling layers and three fully-connected layers.

In transfer learning, the most common approach is copying the first *n* layers of the pre-trained network to the first *n* layers of a target network and then randomly initializing the remaining layers to be trained on the target task. Depending on the size of the target dataset and the number of parameters in the first *n* layers, these copied features can be left unchanged (i.e., frozen) or fine-tuned during the training of the network on a new dataset. It is well accepted that if the target dataset is relatively small, fine-tuning may cause overfitting, whereas if the target dataset is large, then the base features can be fine-tuned to improve the model's performance without overfitting.

**Figure 2:**
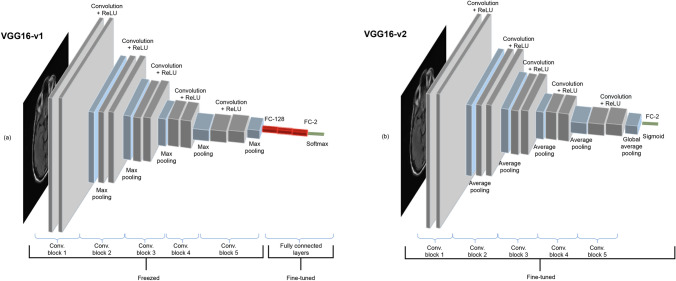
The two different networks based on the VGG16 architecture are shown. Each colored block of layers illustrates a series of convolutions. (a) The first model, named as VGG16-v1 consists of five convolutional blocks followed by three fully connected layers. Only the last three fully connected layers are fine-tuned. (b) On the other hand, the second model, VGG16-v2, has five convolutional blocks followed by a global average pooling layer, and all the layers are fine-tuned.

To investigate the effect of fine-tuning, we have tested two different variants of VGG16 architecture, namely VGG16-v1 and VGG16-v2 (Fig. 2). The former model has been used as a feature extractor where the weights for all network layers are frozen except that of the final fully connected layer. Randomly initialized fully connected layers have replaced the three topmost layers with rectified linear unit (ReLU) activation. The weights are initialized according to the Xavier initialization heuristic^69^ to prevent the gradients from vanishing or exploding.

The VGG16-v2 model has been utilized as a weight initializer where the weights are derived from the pre-trained network and fine-tuned during training. We have replaced the fully connected layers with a randomly initialized global average pooling (GAP) layer suggested by Lin et al.^70^ to reduce the number of parameters and, rather than freezing the CNN layers, we have fine-tuned all layers.

#### ResNet-18 based model

It has been long believed that deeper networks can learn more complex nonlinear relationships than shallower networks with the same number of neurons, and thus network depth is of great importance on model performance^71^. However, many studies revealed that deeper networks often converge at a higher training and test error rate when compared to their shallower counterparts^68^. Therefore, stacking more layers to the plain networks may eventually degrade the model’s performance while complicating the optimization process. To overcome this issue, He and colleagues introduced deep residual neural networks and achieved top-5 test accuracies with their models on the popular ImageNet test set^68^. The model was proposed as an attempt to solve the vanishing gradients and the degradation problems using residual blocks. With these residual blocks, the feature of any deeper unit can be computed as the sum of the activation of a shallower unit and the residual function. This architecture causes the gradient to be directly propagated to shallower units making ResNets easier to train.

**Figure 3:**
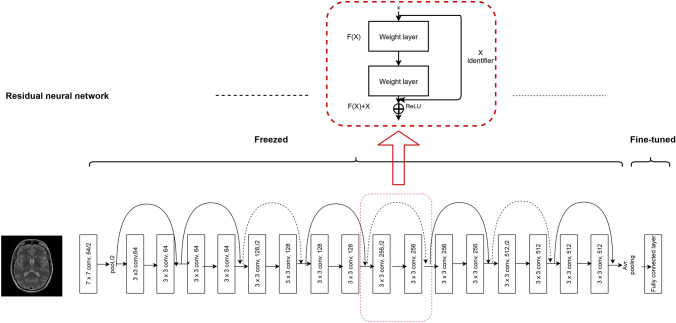
A modified ResNet-18 architecture with an average pooling layer at the end is shown. The upper box represents a residual learning block with an identity shortcut. Each layer is denoted as (filter size, # channels); layers labeled as “freezed” indicates that the weights are not updated during backpropagation, whereas when they are labeled as “fine-tuned” they are updated. The identity shortcuts can be directly used when the input and output are of the same dimensions (solid line shortcuts) and when the dimensions increase (dotted line shortcuts). *ReLU* rectified linear unit.

There are different versions of ResNet architecture with various numbers of layers. In this work, we used ResNet-18 architecture, an 18-layer residual deep learning network consisting of five stages, each with a convolution and identity block^68^. In our model, one fully connected layer with sigmoid activation has been added at the end of the network—a common practice in binary classification tasks as it takes a real-valued input and squashes the output to a range between 0 and 1. Since the network is relatively smaller and has a lower number of parameters than VGG16, the weights and biases of all the transferred layers are fine-tuned while the newly added fully connected layer has been trained to start from randomly initialized weights. The architecture of our ResNet-18 model can be seen in Fig. 3.

### Model training and validation

Each 2D CNN model has been trained and validated using a nested CV strategy—a validation scheme that allows examining the unbiased generalization performance of the trained models along with performing, at the same time, hyperparameters optimization^29^. It involves nesting two *k*-fold CV loops where the inner loop is used for optimizing model hyperparameters, and the outer loop gives an unbiased estimate of the performance of the best model. It is especially suitable when the amount of data available is insufficient to allow separate validation and test sets^29^. A schematic diagram of the procedure is illustrated in Supplementary Fig. S2. It starts by dividing the dataset into *k* folds, and onefold is kept as a test set (outer CV), while the other *k*-1 folds are split into inner folds (inner CV). The model hyperparameters are chosen from the hyperparameter space through a grid search based on the average performance of the model over the inner folds. In particular, we varied the learning rate in the set {10^–5^, 3 × 10^–5^, 10^–4^, 3 × 10^–4^, 10^–3^} and the learning rate decay in {0, 0.1, 0.3, 0.5}. The chosen model is then fitted with all the outer fold training data and tested on the unseen test fold, resulting in an unbiased estimation of the model’s prediction error. Specifically, we choose a tenfold CV because it offers a favorable bias-variance tradeoff^72,73^.

In all experiments, we used batch size = 128 and epoch number = 50. Due to its ability to adaptively updating individual learning rates for each parameter, an Adam optimizer was used^74^. Each selected slice of the 3D T_1_-weighted volume has been classified independently and the final model’s performance was stated using the mean slice-level accuracy, separately, on the training set and test set folds of the outer CV.

We thus conducted CNNs model’s training and validation on each dataset in a nested CV loop using two different data split strategies: (a) subject-level split, in which all the slices of a subject have been placed either in the training set or in the test set, avoiding any form of data leakage; (b) slice-level split, in which all the slices have been pooled together before CV, then split randomly into training and test set. In this case, for each slice of the test set, a set of highly correlated slices coming from the MR volume of the same subject ended up in the training set, giving rise to data leakage, as shown pictographically in Fig. 1.

CNN models were carried out using a custom-made software in Python language (version 3.6.8) using the following modules: CUDA v.9.0.176^75^, TensorFlow-gpu v.1.12.0^76^, Keras v.2.2.4^77^, Scikit-learn v.0.20.2^78^, Nibabel v.2.3.3^79^, and OpenCV v.3.3.0^66^. All the source code can be found in a Github repository at https://github.com/Imaging-AI-for-Health-virtual-lab/Slice-Level-Data-Leakage, and a Docker image can be downloaded at https://hub.docker.com/repository/docker/ai4healthvlab/slice-level-data-leakage. The training and validation of CNN models were performed on a workstation equipped with a 12 GB G5X frame buffer NVIDIA TITAN X (Pascal) GPU with 64 GB RAM, 8 CPUs, 3584 CUDA cores and 11.4 Gbps processing speed. The average computational time for CNN training on a dataset of 34 and 200 subjects were 5.68 h (VGG16-v1), 5.63 h (VGG16-v2), 2.94 h (ResNet-18) and 33.93 h (VGG16-v1), 33.82 h (VGG16-v2), 14.12 h (ResNet-18), respectively. The total computational time for this study was thus about 17 days.

## Supplementary Information

Supplementary Information.
