# Supplementary material for: Effect of data leakage in brain MRI classification using 2D convolutional neural networks
Source: Sci Rep. 2021 Nov 19;11:22544. doi: 10.1038/s41598-021-01681-w (PMC8604922; doi:10.1038/s41598-021-01681-w)
Supplement: Supplementary file 1 — Supplementary Information. [file 41598_2021_1681_MOESM1_ESM.pdf]

# Effect of data leakage in brain MRI classification using 2D convolutional neural networks

Ekin Yagis[1^], Selamawet Workalemahu Atnafu[2^], Alba García Seco de Herrera[1§], Chiara Marzi[2], Riccardo Scheda[2], Marco Giannelli[3], Carlo Tessa[4], Luca Citi[1§], Stefano Diciotti[2§*], for the Alzheimer's Disease Neuroimaging Initiative**

^ *These authors contributed equally to this work*

§ *These authors equally supervised this work*

*** Data used in preparation of this article were obtained from the Alzheimer's Disease Neuroimaging Initiative (ADNI) database (adni.loni.usc.edu). As such, the investigators within the ADNI contributed to the design and implementation of ADNI and/or provided data but did not participate in analysis or writing of this report. A complete listing of ADNI investigators can be found at:*

*http://adni.loni.usc.edu/wp-ontent/uploads/how_to_apply/ADNI_Acknowledgement_List.pdf*

[1] School of Computer Science and Electronic Engineering, University of Essex, Colchester, United Kingdom

[2] Department of Electrical, Electronic, and Information Engineering "Guglielmo Marconi", University of Bologna, Bologna, Italy

[3] Unit of Medical Physics, Pisa University Hospital "Azienda Ospedaliero-Universitaria Pisana", Pisa, Italy

[4] Division of Radiology, Versilia Hospital, Azienda USL Toscana Nord Ovest, Lido di Camaiore (Lu), Italy

**Short title**: Data leakage in brain MRI

*** Corresponding author:** Prof. Stefano Diciotti, Department of Electrical, Electronic, and Information Engineering "Guglielmo Marconi", University of Bologna, Via dell'Università 50, 47521, Cesena, Italy. E-mail: stefano.diciotti@unibo.it

**Supplementary Figure S1.** Sample preprocessed $T_1$-weighted axial images from OASIS-200, ADNI, PPMI and Versilia datasets.

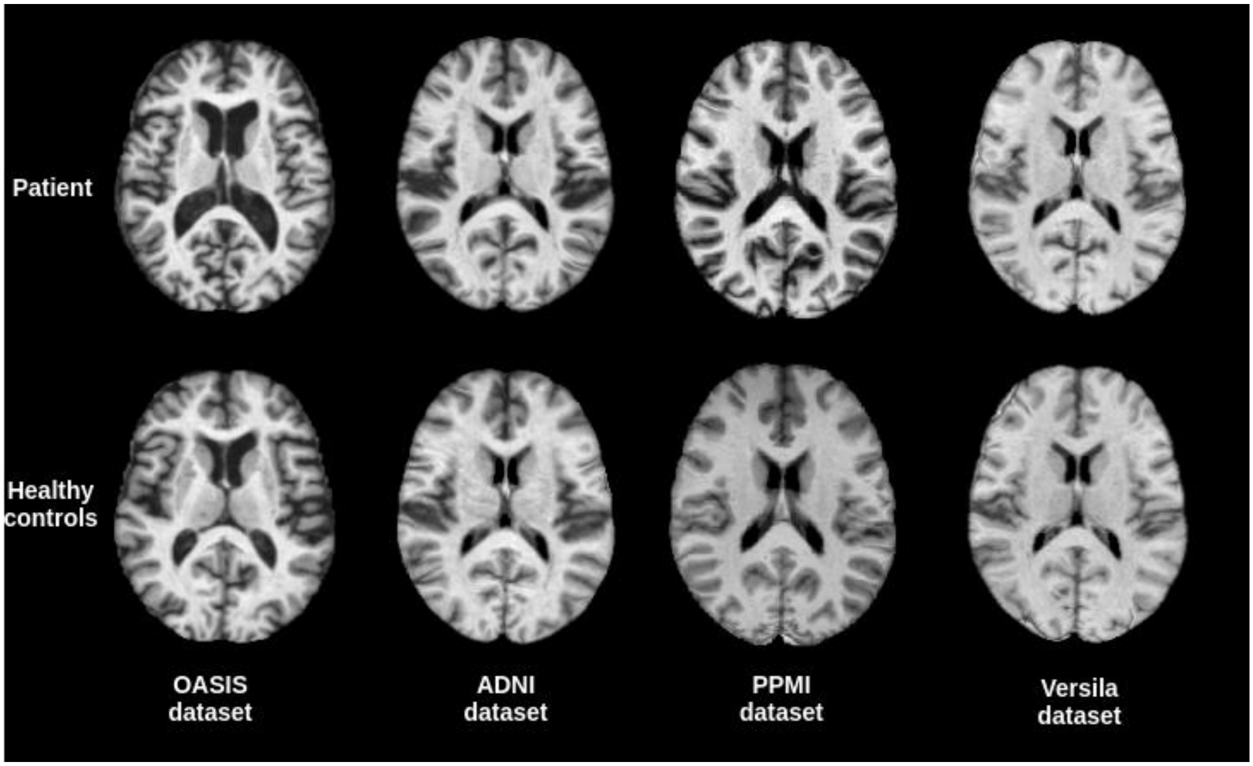

**Supplementary Figure S2.** A scheme of nested CV is represented: the inner CV loop is used to optimize hyperparameters, whereas the outer loop estimates the selected models' performance.

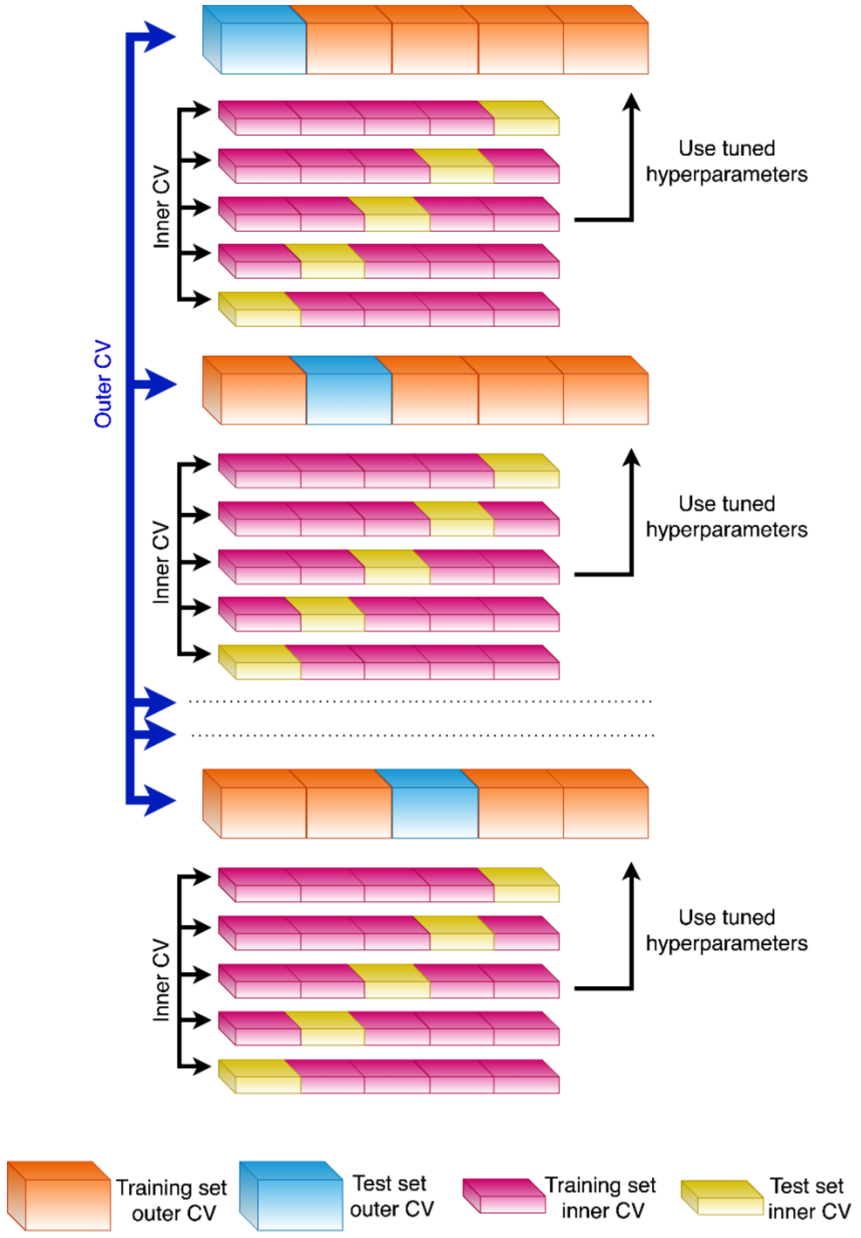

**Supplementary Table S1.** Summary of the previous studies performing classification of neurological disorders using MRI and with clear data leakage.

| Reference | Description |
|---|---|
| Gunawardena et al., 2017[36] | *"The MRI scan produces a 3-dimensional (3D) model of the body. Performing image processing techniques in a 3D MRI film is hard. Therefore it is necessary to convert those 3D MRI films into a series of 2D images before doing any preprocessing [...] Series of 2D images were pre-processed before feature extraction and classification [...] Preprocessed images were further processed in order to achieve the best result. All the images which were to be input to the CNN model were resized into 160 x 160 dimension because different sizes may reduce the accuracy of the classification [...] Afterward, the data set was shuffled. Then the data set has been divided (split) into training set and testing set with a ratio of 80/20 (80% for training and 20% for testing)."* |
| Hon & Khan, 2017[21] | *"Typically, from a 3D MRI scan, we have a large number of images that we can choose from. In most recent methods, the images to be used for training are extracted at random. Instead, in our porposed method, we extract the most informative slices to train the network. For this, we calculate the image entropy of each slice." [...]*<br>*"We used our entropy-based sorting mechanism to pick the most informative 32 images from the axial plane of each 3D scan. That resulted in a total of 6400 training images, 3200 of which were AD and the other 3200 were HC." [...]*<br>*"5-fold cross-validation was used to obtain the results, with an 80% - 20% split between training and testing." [...]*<br>*"in our method, there are total 6,400 images; a 5-fold cross-validation (80% - 20%) split therefore results in a training size of 5,120)." [...]* |
| Jain et al., 2019[37] | *"Brain MR images are in NIfTI format. NIfTI images are volumetric (3D) images, therefore images that we have after pre-processing are all of size 256x256x256. These images comprise of 2D images called slices. Hence, we have 256 slices corresponding to each NIfTI image [...] image entropy based sorting mechanism is used to take most informative slices in which image entropy for each slice was calculated and top 32 slices based on entropy value were selected of each subject [...] Above steps of data processing results in a balanced data-set of 4800 (150 subjects x 32 slices corresponding to each subject) slices which contains 1600 CE, 1600 MCI, and1600 CN slices" [...]*<br>*"Our balanced dataset of 4800 images is shuffled and split into training and test set with split ratio 80:20."* |
| Khagi et al., 2019[38] | *"We have used 28 Normal controls (NC) and 28 Alzheimer's disease (AD) patients for classification, selecting 30 important slices from each patient. Once all the slices are collected, each model was trained, validated and tested in ratio of 6:2:2 on random selection basis."* |
| Sarraf et al., 2017[22] | *"The preprocessed rs-fMRI time series data were first loaded into memory using neuroimaging package Nibabel (http://nipy.org/nibabel/) and were* |

| | |
|---|---|
| | *then decomposed into 2D (x,y) matrices along z and time (t) axes. Next, the 2D matrices were converted to lossless PNG format using the Python OpenCV (opencv.org). The last 10 slices of each time course were removed since they included no functional information. Also, any slices with sum of pixel intensities equal to zero were ignored. During the data conversion process, a total of 793,800 images were produced, including 270,900 Alzheimer's and 522,900 normal control PNG samples [...] The random datasets were labeled for binary classification, and 75% of the images were assigned to the training dataset, while the remaining 25% were used for testing purposes." [...]*<br>*"The preprocessed MRI data were then loaded into memory using a similar approach to the fMRI pipeline and were converted from NII to lossless PNG format using Nibabel and OpenCV, which created two groups (AD and NC) × four preprocessed datasets (MRI 0,2,3,4). Additionally, the slices with zero mean pixels were removed from the data [... ] This step produced a total number of 62,335 images, with 52,507 belonging to the AD group and the remaining 9,828 belonging to the NC group per dataset [...] Next, the model was trained and tested by 75% and 25% of the data"* |
| Wang et al., 2017[39] | Note that in addition to slice-level split, significant data leakage could come from the way augmentation is implemented in this paper. For example, a slice could end up in the training set and a slightly brighter copy of it in the test set.<br><br>*"In this work, we employ the following data augmentation techniques: brightness augmentation, horizontal and vertical shifts, shadow augmentation and flipping." [...]*<br>*"The selected dataset includes serial brain MRI scans from 400 individuals with MCI (age: 74.8±7.4years, 257 Male/143 Female), and 229 healthy elderly controls (age: 76.0±5.0years, 119 Male/110 Female)[...] After data augmentation, we obtain 8000 images including 4000 images of MCI and 4000 images of healthy control. We extract 5000 images for training, 1500 images for validation, 1500 images for testing."* |
| Puranik et al., 2018[40] | *"After the conversion of images to the JPEG format, the last 5 frame images from each time course were scraped as it didn't specifically denote any significant characteristic of the brain. Moreover, the images that were removed were complete black, and would only contribute as noise to the CNN. This generated 474,320 images in all of which 154,000 were Alzheimer disease prone images, 209,440 were normal and 110,880 comprised of EMCI images. These images were pooled together and then randomly shuffled for bifurcation into training and testing dataset in the ratio of 85% and 15% respectively"* |
| Basheera et al., 2019[41] | *"The CNN is used for classification. In our article, we used 224 x 224-sized gray segmented images as input to the CNN." [...]*<br>*"Our total data set has 18,017 GM segmented images. We shuffled and split the data set in the ratio 80:20 as training and test data sets." [...]* |

| | |
|---|---|
| Nawaz et al., 2020[42] | *"Every 3D MRI image contains 256 256 166 slices per volume which cannot be fed to a 2D CNN model. Therefore, we have rescaled each 3D MRI volume and have converted it into 2D slices each of size 300 300 with a single channel for each plane (axial, coronal, sagittal). Each patient contains around 690± 2D slices which can be further fed to train the 2D-CNN model. The pre-processed slices of 3D images are shown in Fig. 1 during different stages." [...]*<br>*"In this paper, we have used 3D structural MRI scans of 160 patients (52 NC, 62 MCI, and 45 AD) to train our 2D-CNN model. The unbalanced (a total of 67413) 2D images are used as a dataset which includes 20972 images for AD class, 26192 images for MCI, and 18513 for NC class. Networks are trained from scratch on data for 70 epochs with a batch size of 100. Experiments are performed using 60% data for training, 20 % for testing, and 20% for the validation set."* Please note that in Table 1 the number of images has been reported. |

**Supplementary Table S2.** Summary of the previous studies performing classification of neurological disorders using MRI and suspected to have potential data leakage.

| Reference | Description |
|---|---|
| Farooq et al., 2017[43] | *"MRI scans are provided in the form of 3D Nifti volumes. At first, skull stripping and gray matter (GM) segmentation is carried out on axial scans through spatial normalization, bias correction and modulation using SPM-8* tool. GM volumes are then converted to JPEG slices using Python Nibabel package. Slices from start and end which contain non information are discarded from the dataset".*<br>Paragraph III.A. *"A subject is scanned at different point of times in different visits, i.e., baseline, after on two and three years. Each such scan is considered as a separate subject in this work. The dataset consists of 33 AD, 22 LMCI, 39 MCI patients and 45 healthy controls which makes a total 355 MRI volumes. Augmentation is done by simply flipping the image along horizontal axis. The balances set includes a total of 9506 images for each class, and a total of 38024 images for all classes". [...]*<br>*"All experiments are performed by splitting data into 25% as test and 75% as train data. 10% data from train set is used as validation set".* |
| Ramzan et al., 2019[44] | *"After applying the preprocessing methods on fMRI data, preprocessed 64×64x48x140 4D fMRI scans are obtained in which each scan contains 64×64x48 3D volumes per time course (140 s). These 4D scans are then converted to 2D images along with image height and time axis. This results in 6720 images of size 64x64 per fMRI scan. The first and last three slices are removed as they contain no functional information. Therefore, from each scan information from 44 slices is used. Hence, 6160 2D images are obtained from each fMRI scan and are saved in portable network graphics (PNG) format. The data acquired from ADNI is processed and converted to 2D images by using the aforementioned pre-processing methods. In this way, we have created a dataset that was used for training deep learning networks." [...]*<br>"*In the dataset, there are 138 4D scans and 850,080 2D images. For the evaluation, we split the dataset into a training dataset, validation dataset and testing dataset with 70%, 20%, and 10% split ratio, respectively as described in Table 6. The dataset was randomly shuffled before splitting.*"<br>Please note that in Table 6, the number of images rather than the number of subjects has been reported for the training, validation, and testing dataset. |
| Raza et al., 2019[45] | *"We used the AlexNet model that takes a 2-d image as an input whereas our brain MRI data is 3-d. Data permutation is used in which multiple slices (Central 20 slices) are extracted from MRI brain data to increase training samples." [...]*<br>*"split ratio for training and test data is set to 0.8 in the experiment. In each plane of OASIS dataset, the number of images for training and testing the classifier are 6656 and 1664 respectively. Similarly, for each plane in ADNI dataset, the number of images for training and testing the classifier is 34912 and 8728 respectively."* |

| | |
|---|---|
| Pathak et al., 2020[46] | *"In our work, we have converted MRI samples into JPEG slices in MATLAB tool. Pixel size of each sample is reduced to 8-bit from 14-bit size by rescaling to 255." [...]*<br>*"Dataset consists of 110 AD, 105 MCI and 51 NC subjects, where each subject contains 44–50 sample of images. Out of which 110 AD subjects are collected from Horizon imaging center [17]. There are total of 9540 images used for training the network and 4193 images for testing. Data augmentation on images is done with rescale operation." [...]*<br>*"We have conducted four experiments of our dataset. For two experiments, as shown in Table 4, 70% of the data was used for training and 30% for validation."* Please note that in Table 4 the number of images rather than the number of subjects has been reported for training and validation.<br>*"Remaining two experiments are conducted with our dataset by removing some blank and unwanted images. In this, 75% of the reduced data was used for training and 25% for validation for remaining two experiments are shown in Table 5."* Please note that also in Table 5 the number of images rather than the number of subjects has been reported for training and validation. |
| Libero et al., 2015[47] | We suspect that feature selection was performed on the whole dataset, before the application of the ML validation scheme.<br><br>*"Nineteen high-functioning adults with ASD (15 males/4 females; mean age: 27.1 years) and 18 typically developing (TD) peers (14 males/4 females; mean age: 24.6 years) participated in this multimodal neuroimaging study (see Table 1 for demographic information)." […]*<br>*"Groups were compared on the resulting cortical thickness values using ANCOVAs conducted using SPSS 22.0 software. Age was used as a covariate for all between-group analyses, as well as average hemispheric cortical thickness." […]*<br>*"1H-MRS ratios were compared using ANCOVA, covarying for age, and GM content." […]*<br>*"To compare the ASD and TD groups on FA, RD, MD, and AD, t- tests were conducted point-wise along each fiber tract for 100 points. A permutation based multiple comparison correction was applied to determine statistical significance (Nichols & Holmes, 2002), p < .05."*<br>*"Leave-one-subject-out cross validation was performed for both regression and classification." […]*<br>*"The data points included were the significant resulting values of the statistical analyses of separate neuroimaging modalities."* |
| Zhou et al., 2014[48] | We suspect that feature selection was performed on the whole dataset, before the application of the ML validation scheme.<br><br>*"To reduce possible classifier overfitting and improve generalization, feature selection was performed in two steps. First, principal component analysis was used to decompose the covariance matrix of the imaging features using the singular value decomposition program in Matlab (release 2010b; MathWorks, Natick, Mass) [33] after variance normalization. Then the number of sorted components based on singular values that contained* |

| | |
|---|---|
| | *99% or 95% of the information from the covariance matrix of all features was determined. Finally, an advanced feature selection algorithm, based on mutual-information and integration of both mRMR criteria [34], was used to select imaging features based on the number of features (components) determined via principal component analysis."* |
| Sivaranjini, et al., 2019[26] | *"The image dataset with 80% of the input data is used for training and the remaining 20% is used for testing. The number of images from each subject given to the deep learning model is averaged to be 40 ± 5 slices based on the selection criterion as shown in Table 2. These images are given to the subsequent convolution layers."* Please note that also in Table 2 the number of images rather than the number of subjects has been reported for training and testing. |
| Lui et al., 2014[49] | We suspect that feature selection was performed on the whole dataset, before the application of the ML validation scheme.<br><br>*"All original features are normalized by removing the mean of each feature and dividing by its SD. We used the feature selection procedure, mRMR,24 to incrementally choose the most representative subset of imaging features, to increase relevance, and decrease redundancy." [...]*<br>*"We used 5 types of mainstream classifiers on the features chosen by mRMR: support vector machine (SVM), naive Bayesian, Bayesian network, radial basis network, and multilayer perceptron [...] We also applied the above methodology to evaluate the achievable performance of different classifiers using the single best feature alone and for mRMR selected features."* |
| Hasan et al., 2019[50] | *"Hasan and Meziane [2] refined these texture measures by ignoring the irrelevant features using analysis of variance method (ANOVA) and reduced to eleven texture measures for each co-occurrence matrix, namely, the contrast, the dissimilarity, the correlation, the sum of square variance, the sum variance, he sum average, the difference entropy, the inverse difference normalized (IDN), the information measure of correlation I (IMC1), the inverse difference moment normalized (IDMN) and the weighted distance in addition to the cross correlation. The total number of texture measures was reduced from 190 to 100 feature measures after using ANOVA." [...]*<br>*"In this study, a total of 6000 MRI axial slices from 600 patients (300 normal, and 300 abnormal) were collected [...] The number of slices for each MRI scan is about 75 slices. [...] The collected MRI dataset is adopted to validate the proposed method. Support vector machine (SVM) with 10-fold cross validation method are applied for accuracy rate estimation of the proposed method. The dataset is divided randomly into 10 folds that are roughly of equal size. Each MRI slice in the given dataset was normalized with 'zero- center' before submission to CNN."* |

**Supplementary Table S3.** Summary of the previous studies performing classification of neurological disorders using MRI and that provide insufficient information to assess data leakage.

| Reference | Description |
|---|---|
| Al-Khuzaie et al., 2021[51] | Section 3 *"Table 1 demonstrates the number of MRI slices."*<br>Section 4 "*The training data set was 75% and the validation data set was 25%."* Please note that also in Figure 3 the input is *"MRI slices dataset"*. |
| Wu et al., 2018[23] | *"Then, from among about 160 slices of raw MR scans of each subject, we discarded the first and last 15 slices without anatomical information, resulting in about 130 slices for each subject. Next, we selected 48 different slices randomly from the remaining slices with the interval of 4, and thus generated 16 RGB color images for each subject. Third, the selected slices were converted into portable network graphics (PNG) format. Finally, all of the RGB color images were resized to 256×256 pixels and converted to the Lightning Memory-Mapped Database (LMDB) for high throughput of the CaffeNet deep learning platform. To ensure the robustness of the model, five random datasets were created to repeat the training and testing of the CNN classifiers (5-fold cross-validation). The flow chart for this is shown as in Figure 4." [...]*<br>*"Differential diagnosis of MCI" "According to aforementioned data augmentation, all baseline MR data were expanded to up to 7,200 slices (4,800 for training, 2,400 for testing) for 150 NC subjects, 7,200 slices (4,800 for training, 2,400 for testing) for 150 patients with sMCI, and 7,536 slices (5,024 for training, 2,512 for testing) for 157 patients with cMCI. During the training model, embedded five-fold cross validation was employed to train a robust model."* |

**Supplementary Table S4.** OASIS-200 is sub-sampled ten times by selecting 34 subjects (17 healthy controls (label=0) and 17 Alzheimer disease patients (label=1)) to produce 10 different OASIS_34 small datasets. The subject IDs and the demographic data associated with each subject are given below. Age is in years. F, female; M, male, OASIS, Open Access Series of Imaging Studies.

| Sub-sample 1 | | | | Sub-sample 2 | | | | Sub-sample 3 | | | | Sub-sample 4 | | | | Sub-sample 5 | | | |
|---|---|---|---|---|---|---|---|---|---|---|---|---|---|---|---|---|---|---|---|
| id | label | sex | age | id | label | sex | age | id | label | sex | age | id | label | sex | age | id | label | sex | age |
| 27 | 0 | F | 82 | 8 | 0 | F | 89 | 97 | 0 | F | 60 | 9 | 0 | M | 89 | 95 | 0 | M | 61 |
| 40 | 0 | F | 78 | 54 | 0 | F | 73 | 58 | 0 | F | 73 | 74 | 0 | M | 69 | 77 | 0 | M | 68 |
| 63 | 0 | M | 71 | 97 | 0 | F | 60 | 68 | 0 | M | 71 | 15 | 0 | M | 87 | 15 | 0 | M | 87 |
| 24 | 0 | F | 83 | 65 | 0 | F | 71 | 74 | 0 | M | 69 | 53 | 0 | M | 74 | 7 | 0 | F | 90 |
| 50 | 0 | F | 74 | 13 | 0 | F | 88 | 2 | 0 | F | 91 | 46 | 0 | M | 75 | 6 | 0 | F | 90 |
| 53 | 0 | M | 74 | 37 | 0 | F | 80 | 46 | 0 | M | 75 | 28 | 0 | F | 81 | 68 | 0 | M | 71 |
| 78 | 0 | M | 68 | 12 | 0 | F | 88 | 88 | 0 | M | 64 | 91 | 0 | M | 62 | 46 | 0 | M | 75 |
| 21 | 0 | M | 84 | 43 | 0 | F | 76 | 26 | 0 | F | 82 | 25 | 0 | F | 83 | 34 | 0 | F | 80 |
| 80 | 0 | F | 67 | 58 | 0 | F | 73 | 56 | 0 | F | 73 | 75 | 0 | F | 69 | 44 | 0 | F | 75 |
| 89 | 0 | F | 64 | 27 | 0 | F | 82 | 18 | 0 | M | 86 | 76 | 0 | F | 69 | 26 | 0 | F | 82 |
| 71 | 0 | M | 70 | 30 | 0 | F | 81 | 19 | 0 | F | 85 | 97 | 0 | F | 60 | 81 | 0 | F | 67 |
| 29 | 0 | F | 81 | 64 | 0 | F | 71 | 49 | 0 | F | 74 | 61 | 0 | F | 72 | 73 | 0 | F | 69 |
| 23 | 0 | F | 84 | 2 | 0 | F | 91 | 75 | 0 | F | 69 | 13 | 0 | F | 88 | 98 | 0 | F | 59 |
| 85 | 0 | F | 65 | 89 | 0 | F | 64 | 87 | 0 | F | 64 | 66 | 0 | F | 71 | 71 | 0 | M | 70 |
| 59 | 0 | F | 73 | 53 | 0 | M | 74 | 72 | 0 | F | 70 | 51 | 0 | F | 74 | 40 | 0 | F | 78 |
| 49 | 0 | F | 74 | 80 | 0 | F | 67 | 61 | 0 | F | 72 | 50 | 0 | F | 74 | 22 | 0 | M | 84 |
| 91 | 0 | M | 62 | 85 | 0 | F | 65 | 0 | 0 | F | 94 | 27 | 0 | F | 82 | 52 | 0 | M | 74 |
| 135 | 1 | F | 80 | 188 | 1 | M | 68 | 173 | 1 | F | 72 | 182 | 1 | M | 70 | 146 | 1 | M | 78 |
| 148 | 1 | F | 77 | 138 | 1 | M | 79 | 176 | 1 | F | 71 | 115 | 1 | M | 84 | 134 | 1 | F | 80 |
| 128 | 1 | M | 81 | 194 | 1 | M | 66 | 188 | 1 | M | 68 | 138 | 1 | M | 79 | 179 | 1 | F | 71 |
| 191 | 1 | F | 67 | 163 | 1 | M | 73 | 127 | 1 | F | 81 | 132 | 1 | F | 80 | 170 | 1 | F | 72 |
| 192 | 1 | F | 66 | 161 | 1 | M | 74 | 116 | 1 | F | 83 | 154 | 1 | F | 76 | 122 | 1 | M | 82 |
| 198 | 1 | F | 63 | 162 | 1 | F | 73 | 197 | 1 | M | 64 | 117 | 1 | F | 83 | 149 | 1 | F | 77 |
| 144 | 1 | F | 78 | 169 | 1 | F | 73 | 198 | 1 | F | 63 | 124 | 1 | F | 81 | 165 | 1 | F | 73 |
| 163 | 1 | M | 73 | 113 | 1 | F | 84 | 118 | 1 | F | 83 | 111 | 1 | M | 86 | 183 | 1 | M | 70 |
| 104 | 1 | M | 90 | 189 | 1 | F | 67 | 168 | 1 | M | 73 | 198 | 1 | F | 63 | 172 | 1 | F | 72 |
| 140 | 1 | F | 78 | 187 | 1 | M | 69 | 134 | 1 | F | 80 | 161 | 1 | M | 74 | 141 | 1 | F | 78 |
| 186 | 1 | F | 69 | 198 | 1 | F | 63 | 147 | 1 | F | 78 | 160 | 1 | F | 74 | 175 | 1 | F | 72 |
| 197 | 1 | M | 64 | 192 | 1 | F | 66 | 106 | 1 | M | 88 | 135 | 1 | F | 80 | 154 | 1 | F | 76 |
| 187 | 1 | M | 69 | 179 | 1 | F | 71 | 157 | 1 | F | 75 | 106 | 1 | M | 88 | 112 | 1 | F | 84 |
| 130 | 1 | M | 80 | 119 | 1 | F | 83 | 107 | 1 | F | 87 | 101 | 1 | F | 92 | 150 | 1 | M | 77 |
| 172 | 1 | F | 72 | 101 | 1 | F | 92 | 146 | 1 | M | 78 | 125 | 1 | M | 81 | 195 | 1 | F | 65 |
| 157 | 1 | F | 75 | 121 | 1 | F | 83 | 196 | 1 | M | 64 | 129 | 1 | F | 80 | 168 | 1 | M | 73 |
| 152 | 1 | M | 77 | 183 | 1 | M | 70 | 181 | 1 | M | 70 | 152 | 1 | M | 77 | 124 | 1 | F | 81 |

| Sub-sample 6 | | | | Sub-sample 7 | | | | Sub-sample 8 | | | | Sub-sample 9 | | | | Sub-sample 10 | | | |
|---|---|---|---|---|---|---|---|---|---|---|---|---|---|---|---|---|---|---|---|
| id | label | sex | age | id | label | sex | age | id | label | sex | age | id | label | sex | age | id | label | sex | age |
| 75 | 0 | F | 69 | 4 | 0 | F | 90 | 24 | 0 | F | 83 | 39 | 0 | F | 78 | 88 | 0 | M | 64 |
| 71 | 0 | M | 70 | 59 | 0 | F | 73 | 89 | 0 | F | 64 | 38 | 0 | F | 78 | 8 | 0 | F | 89 |
| 39 | 0 | F | 78 | 44 | 0 | F | 75 | 33 | 0 | M | 80 | 37 | 0 | F | 80 | 85 | 0 | F | 65 |
| 14 | 0 | F | 88 | 55 | 0 | F | 73 | 31 | 0 | M | 81 | 72 | 0 | F | 70 | 14 | 0 | F | 88 |
| 84 | 0 | F | 65 | 33 | 0 | M | 80 | 55 | 0 | F | 73 | 33 | 0 | M | 80 | 26 | 0 | F | 82 |
| 1 | 0 | F | 93 | 41 | 0 | F | 77 | 67 | 0 | F | 71 | 97 | 0 | F | 60 | 90 | 0 | F | 63 |
| 90 | 0 | F | 63 | 37 | 0 | F | 80 | 66 | 0 | F | 71 | 86 | 0 | M | 65 | 4 | 0 | F | 90 |
| 24 | 0 | F | 83 | 71 | 0 | M | 70 | 83 | 0 | F | 65 | 49 | 0 | F | 74 | 28 | 0 | F | 81 |
| 67 | 0 | F | 71 | 9 | 0 | M | 89 | 98 | 0 | F | 59 | 80 | 0 | F | 67 | 57 | 0 | F | 73 |
| 61 | 0 | F | 72 | 86 | 0 | M | 65 | 88 | 0 | M | 64 | 60 | 0 | F | 72 | 59 | 0 | F | 73 |
| 87 | 0 | F | 64 | 2 | 0 | F | 91 | 40 | 0 | F | 78 | 55 | 0 | F | 73 | 9 | 0 | M | 89 |
| 89 | 0 | F | 64 | 34 | 0 | F | 80 | 51 | 0 | F | 74 | 73 | 0 | F | 69 | 80 | 0 | F | 67 |
| 72 | 0 | F | 70 | 66 | 0 | F | 71 | 7 | 0 | F | 90 | 25 | 0 | F | 83 | 82 | 0 | F | 66 |
| 43 | 0 | F | 76 | 81 | 0 | F | 67 | 86 | 0 | M | 65 | 23 | 0 | F | 84 | 19 | 0 | F | 85 |
| 34 | 0 | F | 80 | 1 | 0 | F | 93 | 68 | 0 | M | 71 | 32 | 0 | F | 80 | 62 | 0 | M | 72 |
| 70 | 0 | F | 70 | 25 | 0 | F | 83 | 95 | 0 | M | 61 | 19 | 0 | F | 85 | 61 | 0 | F | 72 |
| 77 | 0 | M | 68 | 61 | 0 | F | 72 | 54 | 0 | F | 73 | 54 | 0 | F | 73 | 66 | 0 | F | 71 |
| 176 | 1 | F | 71 | 168 | 1 | M | 73 | 156 | 1 | M | 75 | 125 | 1 | M | 81 | 176 | 1 | F | 71 |
| 129 | 1 | F | 80 | 137 | 1 | M | 79 | 117 | 1 | F | 83 | 153 | 1 | M | 76 | 162 | 1 | F | 73 |
| 111 | 1 | M | 86 | 196 | 1 | M | 64 | 164 | 1 | F | 73 | 129 | 1 | F | 80 | 157 | 1 | F | 75 |
| 196 | 1 | M | 64 | 178 | 1 | M | 71 | 148 | 1 | F | 77 | 180 | 1 | M | 71 | 149 | 1 | F | 77 |
| 143 | 1 | M | 78 | 130 | 1 | M | 80 | 159 | 1 | M | 75 | 140 | 1 | F | 78 | 104 | 1 | M | 90 |
| 100 | 1 | F | 96 | 134 | 1 | F | 80 | 155 | 1 | F | 75 | 177 | 1 | M | 71 | 165 | 1 | F | 73 |
| 139 | 1 | F | 79 | 157 | 1 | F | 75 | 187 | 1 | M | 69 | 138 | 1 | M | 79 | 123 | 1 | M | 82 |
| 122 | 1 | M | 82 | 100 | 1 | F | 96 | 124 | 1 | F | 81 | 167 | 1 | F | 73 | 191 | 1 | F | 67 |
| 186 | 1 | F | 69 | 195 | 1 | F | 65 | 129 | 1 | F | 80 | 189 | 1 | F | 67 | 138 | 1 | M | 79 |
| 135 | 1 | F | 80 | 116 | 1 | F | 83 | 193 | 1 | F | 66 | 169 | 1 | F | 73 | 130 | 1 | M | 80 |
| 178 | 1 | M | 71 | 192 | 1 | F | 66 | 163 | 1 | M | 73 | 195 | 1 | F | 65 | 199 | 1 | F | 62 |
| 165 | 1 | F | 73 | 177 | 1 | M | 71 | 132 | 1 | F | 80 | 100 | 1 | F | 96 | 178 | 1 | M | 71 |
| 195 | 1 | F | 65 | 101 | 1 | F | 92 | 162 | 1 | F | 73 | 196 | 1 | M | 64 | 148 | 1 | F | 77 |
| 114 | 1 | M | 84 | 151 | 1 | F | 77 | 194 | 1 | M | 66 | 132 | 1 | F | 80 | 164 | 1 | F | 73 |
| 173 | 1 | F | 72 | 159 | 1 | M | 75 | 169 | 1 | F | 73 | 127 | 1 | F | 81 | 106 | 1 | M | 88 |
| 174 | 1 | F | 72 | 126 | 1 | F | 81 | 190 | 1 | M | 67 | 197 | 1 | M | 64 | 126 | 1 | F | 81 |
| 161 | 1 | M | 74 | 164 | 1 | F | 73 | 127 | 1 | F | 81 | 101 | 1 | F | 92 | 109 | 1 | F | 86 |

**Supplementary Table S5.** Thirty-four subjects (17 AD and 17 HC) have been randomly sampled ten times to produce sub-sampled OASIS-34 datasets. The demographic features of each sub-sampled dataset are listed. Differences between AD and HC groups were assessed through a t-test and a $\chi^2$-test for age and gender, respectively. The p-values are also reported.

| **OASIS subsample** | | **AD patients** | **Healthy controls** | **p-value** |
|---|---|---|---|---|
| Sample-1 | Age (range, years) | 62 – 84 | 63 - 90 | |
| | Age (mean ± SD, years) | 73.7 ± 7.0 | 74.0 ± 6.9 | 0.72 |
| | Gender (women/men) | 11/6 | 10/7 | 0.45 |
| Sample-2 | Age (range, years) | 60 – 91 | 63 – 92 | |
| | Age (mean ± SD, years) | 76.0 ± 9.1 | 73.7 ± 7.6 | 0.02 |
| | Gender (women/men) | 16/1 | 10/7 | 0.22 |
| Sample-3 | Age (range, years) | 60 – 94 | 63 - 88 | |
| | Age (mean ± SD, years) | 74.8 ± 9.3 | 75.1 ± 7.7 | 0.47 |
| | Gender (women/men) | 12/5 | 10/7 | 0.45 |
| Sample-4 | Age (range, years) | 60 – 89 | 63 – 92 | |
| | Age (mean ± SD, years) | 75.2 ± 8.3 | 79.2 ± 6.6 | 0.49 |
| | Gender (women/men) | 11/6 | 9/8 | 0.07 |
| Sample-5 | Age (range, years) | 59 – 90 | 65 – 84 | |
| | Age (mean ± SD, years) | 75.2 ± 9.0 | 75.3 ± 4.8 | 0.29 |
| | Gender (women/men) | 9/8 | 12/5 | 0.49 |
| Sample-6 | Age (range, years) | 63 – 93 | 64 – 96 | |
| | Age (mean ± SD, years) | 73.1 ± 8.4 | 76.2 ± 7.8 | 0.05 |
| | Gender (women/men) | 15/2 | 10/7 | 0.15 |

| | | | | |
|---|---|---|---|---|
| Sample-7 | Age (range, years) | 65 – 93 | 64 – 96 | |
| | Age (mean ± SD, years) | 78.1 ± 8.3 | 76.5 ± 8.4 | 0.27 |
| | Gender (women/men) | 13/4 | 10/7 | 0.29 |
| Sample-8 | Age (range, years) | 59 – 90 | 66 – 83 | |
| | Age (mean ± SD, years) | 71.9 ± 8.2 | 74.5 ± 5.2 | 1.00 |
| | Gender (women/men) | 11/6 | 11/6 | 0.15 |
| Sample-9 | Age (range, years) | 60 – 85 | 64 – 96 | |
| | Age (mean ± SD, years) | 74.7 ± 6.8 | 75.9 ± 8.7 | 0.05 |
| | Gender (women/men) | 15/2 | 10/7 | 0.34 |
| Sample-10 | Age (range, years) | 63 – 90 | 62 – 90 | |
| | Age (mean ± SD, years) | 75.8 ± 9.4 | 76.7 ± 7.1 | 0.24 |
| | Gender (women/men) | 14/3 | 11/6 | 0.38 |

AD = Alzheimer's disease; HC = Healthy controls; OASIS = Open Access Series of Imaging Studies; SD = standard deviation.

**Supplementary Table S6.** Subject IDs and associated demographics for OASIS_200 dataset. The first 100 subjects are from the healthy control group (label = 0) and the last 100 subjects belong to Alzheimer disease patient group (label = 1). Age is in years. F, female; M, male; OASIS, Open Access Series of Imaging Studies.

| OASIS_IDs | NIFTI_IDs | labels | sex | age |
|---|---|---|---|---|
| 221 | 0 | 0 | F | 94 |
| 270 | 1 | 0 | F | 93 |
| 284 | 2 | 0 | F | 91 |
| 65 | 3 | 0 | M | 90 |
| 83 | 4 | 0 | F | 90 |
| 299 | 5 | 0 | F | 90 |
| 301 | 6 | 0 | F | 90 |
| 445 | 7 | 0 | F | 90 |
| 19 | 8 | 0 | F | 89 |
| 32 | 9 | 0 | M | 89 |
| 197 | 10 | 0 | F | 89 |
| 271 | 11 | 0 | F | 89 |
| 169 | 12 | 0 | F | 88 |
| 176 | 13 | 0 | F | 88 |
| 342 | 14 | 0 | F | 88 |
| 260 | 15 | 0 | M | 87 |
| 363 | 16 | 0 | M | 87 |
| 157 | 17 | 0 | F | 86 |
| 317 | 18 | 0 | M | 86 |
| 201 | 19 | 0 | F | 85 |
| 254 | 20 | 0 | F | 85 |
| 110 | 21 | 0 | M | 84 |
| 186 | 22 | 0 | M | 84 |
| 428 | 23 | 0 | F | 84 |
| 75 | 24 | 0 | F | 83 |
| 113 | 25 | 0 | F | 83 |
| 146 | 26 | 0 | F | 82 |
| 426 | 27 | 0 | F | 82 |
| 13 | 28 | 0 | F | 81 |
| 106 | 29 | 0 | F | 81 |
| 228 | 30 | 0 | F | 81 |
| 337 | 31 | 0 | M | 81 |
| 33 | 32 | 0 | F | 80 |
| 138 | 33 | 0 | M | 80 |
| 180 | 34 | 0 | F | 80 |
| 244 | 35 | 0 | F | 80 |

| | | | | |
|---|---|---|---|---|
| 330 | 36 | 0 | F | 80 |
| 446 | 37 | 0 | F | 80 |
| 206 | 38 | 0 | F | 78 |
| 259 | 39 | 0 | F | 78 |
| 280 | 40 | 0 | F | 78 |
| 64 | 41 | 0 | F | 77 |
| 338 | 42 | 0 | M | 77 |
| 195 | 43 | 0 | F | 76 |
| 220 | 44 | 0 | F | 75 |
| 234 | 45 | 0 | M | 75 |
| 423 | 46 | 0 | M | 75 |
| 1 | 47 | 0 | F | 74 |
| 10 | 48 | 0 | M | 74 |
| 165 | 49 | 0 | F | 74 |
| 212 | 50 | 0 | F | 74 |
| 241 | 51 | 0 | F | 74 |
| 354 | 52 | 0 | M | 74 |
| 365 | 53 | 0 | M | 74 |
| 62 | 54 | 0 | F | 73 |
| 279 | 55 | 0 | F | 73 |
| 326 | 56 | 0 | F | 73 |
| 355 | 57 | 0 | F | 73 |
| 369 | 58 | 0 | F | 73 |
| 404 | 59 | 0 | F | 73 |
| 139 | 60 | 0 | F | 72 |
| 237 | 61 | 0 | F | 72 |
| 332 | 62 | 0 | M | 72 |
| 170 | 63 | 0 | M | 71 |
| 203 | 64 | 0 | F | 71 |
| 216 | 65 | 0 | F | 71 |
| 255 | 66 | 0 | F | 71 |
| 341 | 67 | 0 | F | 71 |
| 398 | 68 | 0 | M | 71 |
| 449 | 69 | 0 | F | 71 |
| 85 | 70 | 0 | F | 70 |
| 256 | 71 | 0 | M | 70 |
| 371 | 72 | 0 | F | 70 |
| 112 | 73 | 0 | F | 69 |
| 199 | 74 | 0 | M | 69 |
| 293 | 75 | 0 | F | 69 |
| 422 | 76 | 0 | F | 69 |
| 130 | 77 | 0 | M | 68 |
| 343 | 78 | 0 | M | 68 |

| | | | | |
|---|---|---|---|---|
| 356 | 79 | 0 | F | 68 |
| 68 | 80 | 0 | F | 67 |
| 303 | 81 | 0 | F | 67 |
| 438 | 82 | 0 | F | 66 |
| 30 | 83 | 0 | F | 65 |
| 133 | 84 | 0 | F | 65 |
| 322 | 85 | 0 | F | 65 |
| 358 | 86 | 0 | M | 65 |
| 78 | 87 | 0 | F | 64 |
| 135 | 88 | 0 | M | 64 |
| 292 | 89 | 0 | F | 64 |
| 70 | 90 | 0 | F | 63 |
| 114 | 91 | 0 | M | 62 |
| 457 | 92 | 0 | F | 62 |
| 109 | 93 | 0 | F | 61 |
| 455 | 94 | 0 | F | 61 |
| 456 | 95 | 0 | M | 61 |
| 72 | 96 | 0 | F | 60 |
| 200 | 97 | 0 | F | 60 |
| 289 | 98 | 0 | F | 59 |
| 372 | 99 | 0 | M | 59 |
| 278 | 100 | 1 | F | 96 |
| 400 | 101 | 1 | F | 92 |
| 447 | 102 | 1 | F | 92 |
| 226 | 103 | 1 | M | 90 |
| 247 | 104 | 1 | M | 90 |
| 273 | 105 | 1 | F | 89 |
| 31 | 106 | 1 | M | 88 |
| 137 | 107 | 1 | F | 87 |
| 179 | 108 | 1 | F | 87 |
| 28 | 109 | 1 | F | 86 |
| 351 | 110 | 1 | M | 86 |
| 440 | 111 | 1 | M | 86 |
| 35 | 112 | 1 | F | 84 |
| 161 | 113 | 1 | F | 84 |
| 223 | 114 | 1 | M | 84 |
| 304 | 115 | 1 | M | 84 |
| 53 | 116 | 1 | F | 83 |
| 122 | 117 | 1 | F | 83 |
| 123 | 118 | 1 | F | 83 |
| 286 | 119 | 1 | F | 83 |
| 290 | 120 | 1 | M | 83 |
| 380 | 121 | 1 | F | 83 |

| | | | | |
|---|---|---|---|---|
| 16 | 122 | 1 | M | 82 |
| 23 | 123 | 1 | M | 82 |
| 84 | 124 | 1 | F | 81 |
| 158 | 125 | 1 | M | 81 |
| 164 | 126 | 1 | F | 81 |
| 352 | 127 | 1 | F | 81 |
| 441 | 128 | 1 | M | 81 |
| 21 | 129 | 1 | F | 80 |
| 42 | 130 | 1 | M | 80 |
| 134 | 131 | 1 | M | 80 |
| 166 | 132 | 1 | F | 80 |
| 267 | 133 | 1 | M | 80 |
| 329 | 134 | 1 | F | 80 |
| 335 | 135 | 1 | F | 80 |
| 373 | 136 | 1 | F | 80 |
| 60 | 137 | 1 | M | 79 |
| 263 | 138 | 1 | M | 79 |
| 339 | 139 | 1 | F | 79 |
| 52 | 140 | 1 | F | 78 |
| 185 | 141 | 1 | F | 78 |
| 217 | 142 | 1 | F | 78 |
| 268 | 143 | 1 | M | 78 |
| 287 | 144 | 1 | F | 78 |
| 308 | 145 | 1 | F | 78 |
| 399 | 146 | 1 | M | 78 |
| 425 | 147 | 1 | F | 78 |
| 233 | 148 | 1 | F | 77 |
| 238 | 149 | 1 | F | 77 |
| 315 | 150 | 1 | M | 77 |
| 388 | 151 | 1 | F | 77 |
| 405 | 152 | 1 | M | 77 |
| 15 | 153 | 1 | M | 76 |
| 402 | 154 | 1 | F | 76 |
| 82 | 155 | 1 | F | 75 |
| 205 | 156 | 1 | M | 75 |
| 272 | 157 | 1 | F | 75 |
| 424 | 158 | 1 | M | 75 |
| 452 | 159 | 1 | M | 75 |
| 240 | 160 | 1 | F | 74 |
| 418 | 161 | 1 | M | 74 |
| 3 | 162 | 1 | F | 73 |
| 124 | 163 | 1 | M | 73 |
| 210 | 164 | 1 | F | 73 |

| | | | | |
|---|---|---|---|---|
| 291 | 165 | 1 | F | 73 |
| 312 | 166 | 1 | F | 73 |
| 374 | 167 | 1 | F | 73 |
| 451 | 168 | 1 | M | 73 |
| 454 | 169 | 1 | F | 73 |
| 56 | 170 | 1 | F | 72 |
| 115 | 171 | 1 | M | 72 |
| 269 | 172 | 1 | F | 72 |
| 298 | 173 | 1 | F | 72 |
| 316 | 174 | 1 | F | 72 |
| 432 | 175 | 1 | F | 72 |
| 67 | 176 | 1 | F | 71 |
| 155 | 177 | 1 | M | 71 |
| 288 | 178 | 1 | M | 71 |
| 411 | 179 | 1 | F | 71 |
| 430 | 180 | 1 | M | 71 |
| 39 | 181 | 1 | M | 70 |
| 120 | 182 | 1 | M | 70 |
| 142 | 183 | 1 | M | 70 |
| 453 | 184 | 1 | F | 70 |
| 22 | 185 | 1 | F | 69 |
| 73 | 186 | 1 | F | 69 |
| 390 | 187 | 1 | M | 69 |
| 300 | 188 | 1 | M | 68 |
| 98 | 189 | 1 | F | 67 |
| 307 | 190 | 1 | M | 67 |
| 382 | 191 | 1 | F | 67 |
| 66 | 192 | 1 | F | 66 |
| 94 | 193 | 1 | F | 66 |
| 143 | 194 | 1 | M | 66 |
| 184 | 195 | 1 | F | 65 |
| 46 | 196 | 1 | M | 64 |
| 243 | 197 | 1 | M | 64 |
| 362 | 198 | 1 | F | 63 |
| 41 | 199 | 1 | F | 62 |

**Supplementary Table S7.** Subject IDs and associated demographics for ADNI dataset. The first 100 subjects are from the Alzheimer disease group (label = 1) and the last 100 subjects belong to the healthy control group (label = 0). Age is in years. ADNI, Alzheimer's Disease Neuroimaging Initiative; F, female; M, male.

| ADNI_IDs | NIFTI_IDs | label | age | sex |
|---|---|---|---|---|
| 5275 | 0 | 1 | 78 | F |
| 5006 | 1 | 1 | 68 | F |
| 4252 | 2 | 1 | 87 | F |
| 4338 | 3 | 1 | 81 | M |
| 4990 | 4 | 1 | 75 | F |
| 4756 | 5 | 1 | 84 | M |
| 5029 | 6 | 1 | 80 | M |
| 4954 | 7 | 1 | 61 | M |
| 4774 | 8 | 1 | 86 | M |
| 4195 | 9 | 1 | 62 | M |
| 4124 | 10 | 1 | 72 | M |
| 4672 | 11 | 1 | 67 | M |
| 5163 | 12 | 1 | 67 | M |
| 4615 | 13 | 1 | 87 | M |
| 5149 | 14 | 1 | 84 | M |
| 5087 | 15 | 1 | 65 | F |
| 5027 | 16 | 1 | 76 | M |
| 4537 | 17 | 1 | 77 | F |
| 4039 | 18 | 1 | 56 | M |
| 4625 | 19 | 1 | 64 | M |
| 4879 | 20 | 1 | 80 | F |
| 5162 | 21 | 1 | 69 | M |
| 4732 | 22 | 1 | 77 | M |
| 4993 | 23 | 1 | 72 | F |
| 5013 | 24 | 1 | 68 | F |
| 4968 | 25 | 1 | 79 | M |
| 5206 | 26 | 1 | 85 | M |
| 4845 | 27 | 1 | 68 | F |
| 5016 | 28 | 1 | 64 | F |
| 4280 | 29 | 1 | 80 | M |
| 5090 | 30 | 1 | 59 | M |
| 5184 | 31 | 1 | 73 | F |
| 4024 | 32 | 1 | 56 | F |
| 4001 | 33 | 1 | 89 | F |
| 4905 | 34 | 1 | 73 | F |
| 4894 | 35 | 1 | 61 | F |

| | | | | |
|---|---|---|---|---|
| 5070 | 36 | 1 | 71 | M |
| 5138 | 37 | 1 | 61 | M |
| 5205 | 38 | 1 | 59 | F |
| 4153 | 39 | 1 | 79 | M |
| 4728 | 40 | 1 | 82 | M |
| 5146 | 41 | 1 | 73 | F |
| 4982 | 42 | 1 | 58 | F |
| 4258 | 43 | 1 | 76 | M |
| 5208 | 44 | 1 | 69 | M |
| 4192 | 45 | 1 | 82 | M |
| 4740 | 46 | 1 | 88 | M |
| 4589 | 47 | 1 | 75 | F |
| 5019 | 48 | 1 | 63 | F |
| 5240 | 49 | 1 | 63 | F |
| 4949 | 50 | 1 | 78 | F |
| 5210 | 51 | 1 | 86 | M |
| 4853 | 52 | 1 | 71 | F |
| 5106 | 53 | 1 | 74 | M |
| 4223 | 54 | 1 | 76 | M |
| 5015 | 55 | 1 | 78 | F |
| 5071 | 56 | 1 | 76 | M |
| 4641 | 57 | 1 | 74 | F |
| 4172 | 58 | 1 | 76 | M |
| 4770 | 59 | 1 | 76 | M |
| 4783 | 60 | 1 | 83 | M |
| 4971 | 61 | 1 | 77 | M |
| 5123 | 62 | 1 | 73 | F |
| 4863 | 63 | 1 | 70 | M |
| 4730 | 64 | 1 | 81 | F |
| 4719 | 65 | 1 | 79 | F |
| 4657 | 66 | 1 | 72 | F |
| 4549 | 67 | 1 | 79 | M |
| 4692 | 68 | 1 | 83 | M |
| 4997 | 69 | 1 | 61 | F |
| 4906 | 70 | 1 | 76 | F |
| 5054 | 71 | 1 | 74 | F |
| 4820 | 72 | 1 | 86 | F |
| 5252 | 73 | 1 | 57 | M |
| 4827 | 74 | 1 | 71 | M |
| 5005 | 75 | 1 | 78 | M |
| 4501 | 76 | 1 | 79 | M |
| 4912 | 77 | 1 | 69 | F |
| 4867 | 78 | 1 | 75 | M |

| | | | | |
|---|---|---|---|---|
| 4546 | 79 | 1 | 71 | M |
| 4526 | 80 | 1 | 80 | M |
| 5241 | 81 | 1 | 88 | M |
| 5017 | 82 | 1 | 84 | M |
| 4110 | 83 | 1 | 79 | F |
| 4733 | 84 | 1 | 75 | M |
| 4792 | 85 | 1 | 80 | M |
| 4696 | 86 | 1 | 73 | F |
| 4209 | 87 | 1 | 78 | F |
| 5074 | 88 | 1 | 75 | F |
| 5231 | 89 | 1 | 74 | F |
| 4477 | 90 | 1 | 82 | F |
| 4660 | 91 | 1 | 77 | F |
| 4859 | 92 | 1 | 72 | M |
| 5037 | 93 | 1 | 67 | M |
| 5112 | 94 | 1 | 75 | F |
| 4755 | 95 | 1 | 72 | M |
| 4772 | 96 | 1 | 79 | F |
| 5018 | 97 | 1 | 73 | M |
| 5059 | 98 | 1 | 72 | M |
| 4994 | 99 | 1 | 85 | M |
| 4075 | 100 | 0 | 73 | M |
| 4266 | 101 | 0 | 70 | F |
| 4348 | 102 | 0 | 66 | F |
| 56 | 103 | 0 | 78 | F |
| 4388 | 104 | 0 | 67 | M |
| 89 | 105 | 0 | 71 | M |
| 4739 | 106 | 0 | 65 | M |
| 4071 | 107 | 0 | 85 | M |
| 4150 | 108 | 0 | 74 | M |
| 416 | 109 | 0 | 82 | F |
| 4262 | 110 | 0 | 73 | F |
| 4083 | 111 | 0 | 85 | M |
| 4080 | 112 | 0 | 79 | F |
| 4545 | 113 | 0 | 67 | F |
| 23 | 114 | 0 | 78 | M |
| 4643 | 115 | 0 | 65 | F |
| 4382 | 116 | 0 | 76 | F |
| 59 | 117 | 0 | 79 | F |
| 257 | 118 | 0 | 86 | F |
| 4093 | 119 | 0 | 70 | F |
| 4616 | 120 | 0 | 85 | M |
| 4345 | 121 | 0 | 70 | M |

| | | | | |
|---|---|---|---|---|
| 677 | 122 | 0 | 81 | M |
| 4389 | 123 | 0 | 81 | M |
| 4393 | 124 | 0 | 74 | M |
| 4399 | 125 | 0 | 78 | F |
| 4313 | 126 | 0 | 77 | F |
| 4577 | 127 | 0 | 85 | M |
| 4032 | 128 | 0 | 70 | F |
| 4021 | 129 | 0 | 67 | M |
| 4082 | 130 | 0 | 76 | M |
| 4060 | 131 | 0 | 85 | M |
| 4339 | 132 | 0 | 84 | M |
| 4349 | 133 | 0 | 71 | F |
| 4277 | 134 | 0 | 72 | F |
| 4340 | 135 | 0 | 67 | F |
| 4208 | 136 | 0 | 78 | M |
| 4278 | 137 | 0 | 75 | M |
| 4391 | 138 | 0 | 75 | M |
| 4856 | 139 | 0 | 65 | F |
| 4357 | 140 | 0 | 74 | F |
| 4158 | 141 | 0 | 84 | M |
| 4304 | 142 | 0 | 75 | M |
| 4104 | 143 | 0 | 72 | M |
| 4580 | 144 | 0 | 70 | F |
| 4448 | 145 | 0 | 64 | F |
| 4270 | 146 | 0 | 75 | F |
| 4795 | 147 | 0 | 61 | M |
| 842 | 148 | 0 | 79 | M |
| 4264 | 149 | 0 | 74 | F |
| 311 | 150 | 0 | 83 | F |
| 4086 | 151 | 0 | 82 | M |
| 4010 | 152 | 0 | 71 | F |
| 4367 | 153 | 0 | 65 | F |
| 4222 | 154 | 0 | 82 | F |
| 4386 | 155 | 0 | 85 | F |
| 5023 | 156 | 0 | 64 | F |
| 4218 | 157 | 0 | 81 | M |
| 4878 | 158 | 0 | 73 | F |
| 4120 | 159 | 0 | 82 | F |
| 4076 | 160 | 0 | 73 | F |
| 685 | 161 | 0 | 95 | F |
| 21 | 162 | 0 | 79 | F |
| 4257 | 163 | 0 | 79 | M |
| 4291 | 164 | 0 | 76 | F |

| | | | | |
|---|---|---|---|---|
| 4612 | 165 | 0 | 69 | F |
| 4559 | 166 | 0 | 67 | F |
| 4308 | 167 | 0 | 74 | M |
| 4762 | 168 | 0 | 74 | M |
| 454 | 169 | 0 | 89 | F |
| 4196 | 170 | 0 | 79 | M |
| 4084 | 171 | 0 | 68 | F |
| 555 | 172 | 0 | 87 | M |
| 4552 | 173 | 0 | 63 | M |
| 4505 | 174 | 0 | 80 | F |
| 4410 | 175 | 0 | 69 | F |
| 4200 | 176 | 0 | 70 | F |
| 4576 | 177 | 0 | 71 | F |
| 4320 | 178 | 0 | 71 | F |
| 4164 | 179 | 0 | 73 | M |
| 4173 | 180 | 0 | 70 | F |
| 4424 | 181 | 0 | 66 | F |
| 4043 | 182 | 0 | 82 | M |
| 4026 | 183 | 0 | 74 | M |
| 4453 | 184 | 0 | 66 | M |
| 4028 | 185 | 0 | 64 | F |
| 4642 | 186 | 0 | 58 | F |
| 69 | 187 | 0 | 81 | M |
| 4092 | 188 | 0 | 82 | F |
| 4511 | 189 | 0 | 70 | M |
| 4491 | 190 | 0 | 84 | M |
| 473 | 191 | 0 | 83 | M |
| 210 | 192 | 0 | 83 | F |
| 4041 | 193 | 0 | 78 | F |
| 4014 | 194 | 0 | 81 | M |
| 751 | 195 | 0 | 77 | M |
| 4225 | 196 | 0 | 70 | M |
| 498 | 197 | 0 | 80 | M |
| 4037 | 198 | 0 | 76 | M |
| 4337 | 199 | 0 | 72 | M |

**Supplementary Table S8**. Subject IDs and associated demographics for PPMI dataset. The first 100 subjects are from the Parkinson's disease patient group (label = 1) and the last 100 subjects belong to the healthy control group (label = 0). Age is in years. F, female; M, male, PPMI, Parkinson's Progression Markers Initiative.

| PPMI_IDs | NIFTI_IDs | label | sex | age |
|---|---|---|---|---|
| 3625 | 0 | 1 | F | 67 |
| 3060 | 1 | 1 | M | 75 |
| 3577 | 2 | 1 | M | 68 |
| 3830 | 3 | 1 | F | 52 |
| 3709 | 4 | 1 | M | 69 |
| 3591 | 5 | 1 | M | 63 |
| 3154 | 6 | 1 | F | 73 |
| 3814 | 7 | 1 | M | 67 |
| 3056 | 8 | 1 | M | 56 |
| 3327 | 9 | 1 | F | 54 |
| 3176 | 10 | 1 | M | 62 |
| 3229 | 11 | 1 | M | 73 |
| 3770 | 12 | 1 | F | 55 |
| 4099 | 13 | 1 | F | 60 |
| 4102 | 14 | 1 | M | 69 |
| 4038 | 15 | 1 | F | 71 |
| 4071 | 16 | 1 | M | 58 |
| 3575 | 17 | 1 | M | 61 |
| 3771 | 18 | 1 | F | 75 |
| 3003 | 19 | 1 | F | 57 |
| 3364 | 20 | 1 | F | 39 |
| 3608 | 21 | 1 | M | 46 |
| 3116 | 22 | 1 | M | 65 |
| 3522 | 23 | 1 | M | 54 |
| 3288 | 24 | 1 | F | 47 |
| 3632 | 25 | 1 | M | 55 |
| 3309 | 26 | 1 | F | 54 |
| 3150 | 27 | 1 | F | 57 |
| 3970 | 28 | 1 | M | 67 |
| 3638 | 29 | 1 | M | 66 |
| 3232 | 30 | 1 | F | 68 |
| 3454 | 31 | 1 | F | 57 |
| 3616 | 32 | 1 | M | 78 |
| 3455 | 33 | 1 | M | 67 |
| 3023 | 34 | 1 | F | 71 |
| 3083 | 35 | 1 | F | 66 |
| 3325 | 36 | 1 | F | 67 |
| 3218 | 37 | 1 | M | 64 |
| 3429 | 38 | 1 | M | 65 |
| 3653 | 39 | 1 | F | 80 |
| 3514 | 40 | 1 | M | 71 |
| 3119 | 41 | 1 | M | 64 |
| 3752 | 42 | 1 | M | 52 |
| 4022 | 43 | 1 | M | 48 |
| 4122 | 44 | 1 | M | 64 |
| 3436 | 45 | 1 | M | 51 |

| | | | | |
|---|---|---|---|---|
| 3207 | 46 | 1 | F | 58 |
| 3439 | 47 | 1 | M | 57 |
| 3067 | 48 | 1 | M | 74 |
| 3066 | 49 | 1 | F | 64 |
| 3290 | 50 | 1 | M | 63 |
| 3230 | 51 | 1 | M | 70 |
| 3787 | 52 | 1 | M | 49 |
| 4115 | 53 | 1 | M | 67 |
| 3311 | 54 | 1 | M | 75 |
| 3634 | 55 | 1 | M | 43 |
| 3077 | 56 | 1 | M | 63 |
| 3417 | 57 | 1 | M | 57 |
| 3822 | 58 | 1 | M | 56 |
| 4092 | 59 | 1 | F | 77 |
| 3021 | 60 | 1 | F | 64 |
| 4034 | 61 | 1 | F | 55 |
| 3958 | 62 | 1 | M | 76 |
| 4113 | 63 | 1 | F | 34 |
| 3630 | 64 | 1 | F | 61 |
| 3588 | 65 | 1 | F | 49 |
| 3621 | 66 | 1 | F | 54 |
| 3473 | 67 | 1 | F | 55 |
| 3584 | 68 | 1 | M | 43 |
| 3102 | 69 | 1 | M | 64 |
| 3819 | 70 | 1 | F | 53 |
| 3442 | 71 | 1 | M | 63 |
| 3472 | 72 | 1 | M | 61 |
| 4035 | 73 | 1 | M | 60 |
| 3815 | 74 | 1 | M | 62 |
| 3432 | 75 | 1 | M | 64 |
| 3838 | 76 | 1 | F | 61 |
| 4077 | 77 | 1 | M | 48 |
| 3282 | 78 | 1 | F | 62 |
| 3190 | 79 | 1 | M | 82 |
| 3307 | 80 | 1 | M | 66 |
| 3710 | 81 | 1 | M | 56 |
| 3462 | 82 | 1 | F | 44 |
| 3802 | 83 | 1 | M | 70 |
| 3433 | 84 | 1 | F | 82 |
| 3128 | 85 | 1 | F | 60 |
| 3132 | 86 | 1 | M | 50 |
| 3080 | 87 | 1 | M | 80 |
| 3186 | 88 | 1 | F | 62 |
| 4078 | 89 | 1 | M | 70 |
| 3589 | 90 | 1 | F | 75 |
| 3666 | 91 | 1 | M | 52 |
| 3001 | 92 | 1 | M | 65 |
| 3631 | 93 | 1 | F | 68 |
| 3205 | 94 | 1 | M | 73 |
| 3006 | 95 | 1 | F | 58 |
| 3434 | 96 | 1 | M | 54 |
| 3220 | 97 | 1 | F | 74 |
| 3461 | 98 | 1 | M | 63 |

| | | | | |
|---|---|---|---|---|
| 3961 | 99 | 1 | M | 37 |
| 3515 | 100 | 0 | F | 74 |
| 3468 | 101 | 0 | M | 57 |
| 3809 | 102 | 0 | F | 53 |
| 3277 | 103 | 0 | M | 66 |
| 4010 | 104 | 0 | M | 42 |
| 3216 | 105 | 0 | F | 52 |
| 3350 | 106 | 0 | M | 79 |
| 3390 | 107 | 0 | M | 66 |
| 3544 | 108 | 0 | M | 70 |
| 3527 | 109 | 0 | M | 62 |
| 3851 | 110 | 0 | F | 54 |
| 3959 | 111 | 0 | M | 73 |
| 3464 | 112 | 0 | M | 51 |
| 3767 | 113 | 0 | F | 53 |
| 3257 | 114 | 0 | F | 53 |
| 3480 | 115 | 0 | F | 72 |
| 3952 | 116 | 0 | F | 69 |
| 3967 | 117 | 0 | M | 57 |
| 3270 | 118 | 0 | M | 55 |
| 3424 | 119 | 0 | F | 64 |
| 4116 | 120 | 0 | M | 65 |
| 3000 | 121 | 0 | F | 69 |
| 3016 | 122 | 0 | M | 57 |
| 3779 | 123 | 0 | M | 56 |
| 3813 | 124 | 0 | M | 65 |
| 3806 | 125 | 0 | F | 59 |
| 3029 | 126 | 0 | M | 66 |
| 3526 | 127 | 0 | M | 61 |
| 3619 | 128 | 0 | F | 32 |
| 3151 | 129 | 0 | M | 58 |
| 3114 | 130 | 0 | F | 64 |
| 3301 | 131 | 0 | M | 52 |
| 4004 | 132 | 0 | F | 65 |
| 3479 | 133 | 0 | M | 58 |
| 3570 | 134 | 0 | M | 72 |
| 3853 | 135 | 0 | M | 47 |
| 3636 | 136 | 0 | M | 64 |
| 3161 | 137 | 0 | M | 45 |
| 3310 | 138 | 0 | M | 65 |
| 3201 | 139 | 0 | F | 65 |
| 3013 | 140 | 0 | F | 79 |
| 4104 | 141 | 0 | M | 66 |
| 3074 | 142 | 0 | M | 31 |
| 3053 | 143 | 0 | M | 69 |
| 3611 | 144 | 0 | F | 42 |
| 3478 | 145 | 0 | M | 77 |
| 3169 | 146 | 0 | M | 57 |
| 3215 | 147 | 0 | F | 70 |
| 4079 | 148 | 0 | M | 63 |
| 3157 | 149 | 0 | F | 64 |
| 4090 | 150 | 0 | M | 57 |
| 3428 | 151 | 0 | F | 58 |

| | | | | |
|---|---|---|---|---|
| 3206 | 152 | 0 | F | 31 |
| 3368 | 153 | 0 | F | 53 |
| 3355 | 154 | 0 | M | 32 |
| 3405 | 155 | 0 | F | 64 |
| 3160 | 156 | 0 | M | 80 |
| 3361 | 157 | 0 | F | 56 |
| 3613 | 158 | 0 | F | 56 |
| 3320 | 159 | 0 | M | 56 |
| 3411 | 160 | 0 | M | 41 |
| 3519 | 161 | 0 | M | 74 |
| 3008 | 162 | 0 | F | 82 |
| 3969 | 163 | 0 | F | 81 |
| 3358 | 164 | 0 | M | 49 |
| 3362 | 165 | 0 | F | 42 |
| 3219 | 166 | 0 | M | 70 |
| 3759 | 167 | 0 | F | 54 |
| 4085 | 168 | 0 | M | 67 |
| 4032 | 169 | 0 | M | 68 |
| 3551 | 170 | 0 | M | 64 |
| 4118 | 171 | 0 | F | 68 |
| 3615 | 172 | 0 | M | 66 |
| 3965 | 173 | 0 | M | 83 |
| 3064 | 174 | 0 | F | 60 |
| 3057 | 175 | 0 | F | 60 |
| 3807 | 176 | 0 | F | 73 |
| 3075 | 177 | 0 | M | 76 |
| 4139 | 178 | 0 | M | 81 |
| 3466 | 179 | 0 | M | 48 |
| 3410 | 180 | 0 | M | 74 |
| 3523 | 181 | 0 | M | 64 |
| 3768 | 182 | 0 | M | 60 |
| 3651 | 183 | 0 | M | 77 |
| 3004 | 184 | 0 | M | 59 |
| 3115 | 185 | 0 | M | 61 |
| 3855 | 186 | 0 | F | 49 |
| 3156 | 187 | 0 | M | 70 |
| 3453 | 188 | 0 | F | 60 |
| 3525 | 189 | 0 | M | 56 |
| 3852 | 190 | 0 | M | 77 |
| 3071 | 191 | 0 | M | 72 |
| 3521 | 192 | 0 | M | 65 |
| 3955 | 193 | 0 | M | 54 |
| 3656 | 194 | 0 | M | 79 |
| 3554 | 195 | 0 | M | 75 |
| 4105 | 196 | 0 | M | 67 |
| 3859 | 197 | 0 | M | 60 |
| 3817 | 198 | 0 | M | 74 |
| 3457 | 199 | 0 | F | 63 |
